# Extinction blunts paraventricular thalamic contributions to heroin relapse

**DOI:** 10.1016/j.celrep.2021.109605

**Published:** 2021-08-24

**Authors:** Giuseppe Giannotti, Sheng Gong, Nicholas Fayette, Jasper A. Heinsbroek, James E. Orfila, Paco S. Herson, Christopher P. Ford, Jamie Peters

**Affiliations:** 1 Department of Neuroscience, Medical University of South Carolina, Charleston, SC 29425, USA; 2 Department of Anesthesiology, University of Colorado Anschutz Medical Campus, Aurora, CO 80045, USA; 3 Department of Pharmacology, University of Colorado Anschutz Medical Campus, Aurora, CO 80045, USA; 4 Lead contact

## Abstract

Here, we use optogenetics and chemogenetics to investigate the contribution of the paraventricular thalamus (PVT) to nucleus accumbens (NAc) pathway in aversion and heroin relapse in two different heroin self-administration models in rats. In one model, rats undergo forced abstinence in the home cage prior to relapse testing, and in the other, they undergo extinction training, a procedure that is likened to cognitive behavioral therapy. We find that the PVT→NAc pathway is both sufficient and necessary to drive aversion and heroin seeking after abstinence, but not extinction. The ability of extinction to reduce this pathway’s contribution to heroin relapse is accompanied by a loss of synaptic plasticity in PVT inputs onto a specific subset of NAc neurons. Thus, extinction may exert therapeutic reductions in opioid seeking by altering synaptic plasticity within the PVT→NAc pathway, resulting in reduced aversion during opioid withdrawal as well as reduced relapse propensity.

## In brief

Giannotti et al. show that the paraventricular thalamus (PVT) to nucleus accumbens (NAc) pathway drives heroin relapse after abstinence, but not after extinction. Extinction causes a loss of synaptic plasticity in PVT→NAc dopamine D1 receptor expressing NAc medium spiny neurons (D1-MSNs), thereby diminishing the PVT→NAc drive to relapse.

## Graphical Abstract

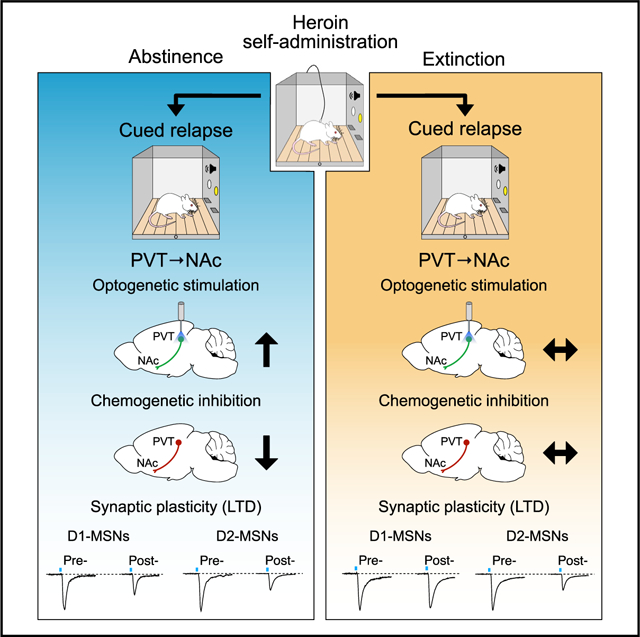

## INTRODUCTION

The paraventricular thalamus (PVT) has been recently identified as a key component in the neural circuitry of drug addiction in both humans and rodents (Huang et al., 2018; McGinty and Otis, 2020; Zhou et al., 2021; Zhou and Zhu, 2019). The PVT interacts with other circuit components involved in reward, emotion, and decision-making (McNally, 2021; Millan et al., 2017), making the PVT a central hub that integrates cortical and subcortical inputs to regulate diverse behavioral responses (Kirouac, 2015; Millan et al., 2017; Penzo and Gao, 2021). Most PVT neurons project to the nucleus accumbens (NAc) (Li and Kirouac, 2008), and activating this output pathway drives aversion in naive animals in real-time conditioned place aversion (rtCPA) tests (Do-Monte et al., 2017; Zhu et al., 2016). Moreover, the PVT→NAc pathway mediates withdrawal signs in morphine-dependent mice (Zhu et al., 2016) and is necessary for retrieval of opioid-associated memories (Keyes et al., 2020). These data support previous findings implicating the PVT in aversive states (Kirouac, 2015, 2021; Li et al., 2011; Yasoshima et al., 2007), and they identify a specific output (to NAc) by which the PVT mediates aversion and morphine-associated memories (Keyes et al., 2020; Zhu et al., 2016).

In preclinical studies, relapse has been primarily investigated using self-administration models wherein animals regulate their own intake of drug in a contingent manner. Two of the most commonly used models of this type employ either a period of home cage abstinence during drug withdrawal or extinction training (Reiner et al., 2019; Venniro et al., 2016). When home cage abstinence is used, a phenomenon known as incubation of craving occurs with increasing withdrawal duration (Pickens et al., 2011). In contrast, extinction training involves new learning that the drug is no longer available contingent on the animals’ response, and thus drug seeking diminishes during the course of withdrawal, eventually reaching very low levels. Relapse can be triggered in either of these models by re-exposing the animals to drug-paired cues that were unavailable during the withdrawal period (Reiner et al., 2019; Venniro et al., 2016). Importantly, evidence suggests the neural circuitry underlying these two forms of relapse is different. For instance, the central nucleus of the amygdala is critical for relapse after abstinence (Venniro et al., 2017, 2020), whereas the prefrontal cortex is recruited by extinction learning to regulate subcortical targets that drive drug seeking (Peters et al., 2009).

We set out to examine the role of the PVT→NAc pathway in aversion and relapse using two different heroin self-administration models in rats to directly compare relapse after abstinence versus extinction. To do this, we used optogenetics and chemogenetics to manipulate activity in the PVT→NAc pathway and investigate whether it is sufficient and/or necessary for these opioid-related behaviors. We found that optogenetic activation of the PVT→NAc pathway is capable of inducing aversion (measured by rtCPA) in animals with a heroin self-administration history, but only after abstinence and not extinction. Similarly, these same optogenetic parameters potentiate heroin relapse after abstinence, but not extinction. To investigate a potential circuit mechanism responsible for these distinct behavioral results, we investigated the synaptic plasticity within the PVT→NAc pathway following abstinence or extinction. We found that long-term depression (LTD) was occluded in a specific subpopulation of NAc neurons that receive input from PVT following extinction. Chemogenetic inhibition of the PVT→NAc pathway, which is hypothesized to emulate extinction-induced plasticity acutely, reduced relapse after abstinence, but not extinction. These data suggest that the contribution of the PVT→NAc pathway to heroin relapse is fundamentally altered by extinction and identify a potential synaptic mechanism by which extinction training blunts the contribution of this pathway to relapse.

## RESULTS

### Validation of the combinatorial virus approach and optogenetic parameters

**Figure 1.**
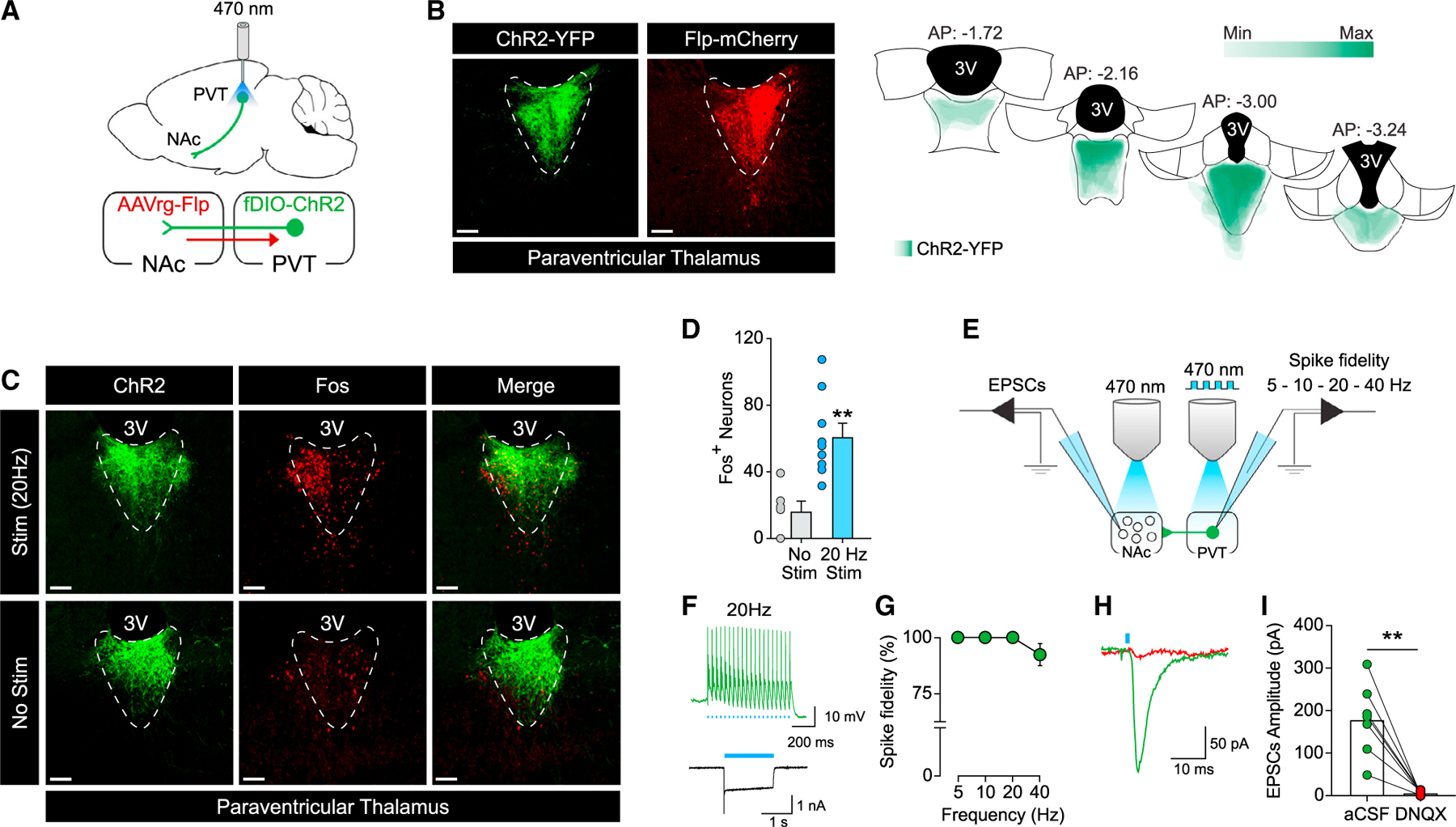
Validation of the combinatorial virus approach and optogenetic parameters (A) Schematic illustration of combinatorial virus approach and optogenetic approach used to target the PVT→NAc pathway. (B) Representative images (left) of ChR2-eYFP (green) and Flp-mCherry (red) expression in the PVT neurons. Placement map (right) shows ChR2-YFP spread in the PVT (each animal represented at 10% opacity). (C) Optogenetic stimulation (stim, 20 Hz, 20 ms) of ChR2-expressing neuron in the PVT induced Fos (top) compared to the non-stimulation condition (no stim, bottom). (D) Quantification of Fos-positive cells in PVT neurons (no stim, n = 6; stim, n = 9; **p < 0.01 versus no stim condition). (E) Schematic illustration of the slice electrophysiology approach employed to investigate ChR2 functionality in the PVT neurons (left) and functional connectivity of the PVT→NAc pathway (right). (F) Example traces of photo-evoked (20 Hz) action potentials (top) and photocurrent (bottom) in ChR2-expressing PVT neurons. (G) Quantification of light-evoked action potential firing at different frequencies of light stimulation (5, 10, 20, and 40 Hz) in ChR2-expressing PVT neurons (n = 11 cells/3 rats). (H and I) Example trace (H) and quantification (I) of EPSCs recorded from NAc neurons after optical stimulation of ChR2-expressing PVT terminals in NAc before and after bath application of DNQX (n = 7 cells/3 rats; **p < 0.01 versus aCSF). Scale bars, 50 μm. Data are shown as mean ± SEM.

We used a retrograde AAVrg-Flp in the NAc, in combination with a Flp-dependent fDIO-ChR2 in the PVT to restrict expression of the ChR2 to the PVT→NAc pathway (Figure 1A). The optogenetic probe was implanted above the PVT for cell-body stimulation. It has been shown that PVT projections to NAc collateralize to different brain regions (Dong et al., 2017). Accordingly, a potential limitation of this approach is that activation at the level of PVT cell bodies may activate collaterals from this pathway as well. Importantly, however, note that collateralized projections are likely to be activated when PVT→NAc neurons fire endogenously. ChR2-eYFP and Flp-mCherry were verified to be confined to the PVT (Figure 1B). Expression analyses for Fos, a marker of neuronal activity, were used to validate the optogenetics approach in the PVT→NAc pathway after light (470 nm) stimulation (stim, 20 Hz, 20 ms – 20 min) or no light control (no stim) (Figure 1C). Robust Fos expression in PVT→NAc neurons was observed in stim compared to no stimulation controls (Figure 1D; t_(13)_ = 3.973, p = 0.0016).

Slice electrophysiology was used to validate the functionality of the ChR2 in PVT neurons and the functional connectivity of the PVT→NAc pathway (Figure 1E). Figure 1F shows example traces of photo-evoked action potentials and photocurrent elicited by light stimulation (470 nm, 20 Hz) of ChR2-expressing PVT neurons. Quantification of action potential firing evoked by light stimulation (5, 10, 20, and 40 Hz) in ChR2-expressing PVT neurons indicated high spike fidelity at the chosen (470 nm, 20 Hz) parameters for behavioral experiments (Figure 1G). Functional connectivity of the PVT→NAc pathway was investigated by analyzing photo-evoked excitatory postsynaptic currents (EPSCs) generated by stimulating ChR2-expressing PVT terminals onto NAc medium spiny neurons (MSNs). Robust, glutamatergic photo-evoked EPSCs were apparent in NAc MSNs (Figure 1H). These PVT-derived EPSCs in NAc MSNs were blocked by bath application of 6,7-dinitroquinoxaline-2,3-dione (DNQX) (Figure 1I; t_(6)_ = 5.383, p = 0.0017).

### Optogenetic activation of the PVT→NAc pathway drives aversion and heroin relapse

**Figure 2.**
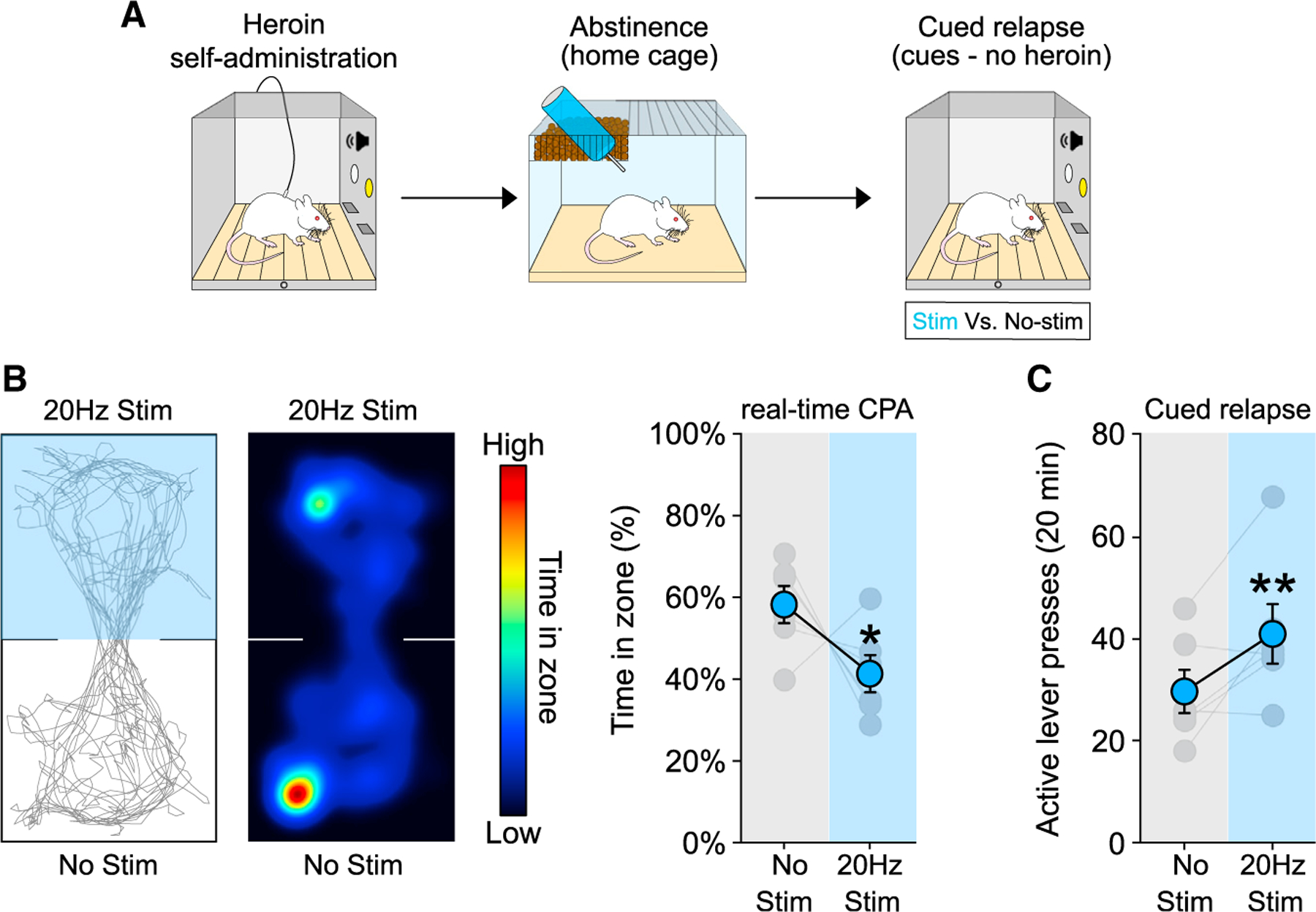
Optogenetic activation of the PVT→NAc pathway induces real-time CPA and drives heroin relapse after abstinence from heroin self-administration (A) Timeline employed for the behavioral experiments. (B and C) Example trace (left) and heatmap (middle) of real-time CPA test after 14 d of abstinence from heroin self-administration (B). Optogenetic activation of the PVT→NAc pathway induces aversion in real-time CPA (B, right; n = 6) and drives heroin relapse (C; n = 6) after 14 days of abstinence. *p < 0.05, **p < 0.01 versus no stim condition. Data are shown as mean ± SEM.

First, we investigated whether optogenetic activation of the PVT→NAc pathway was capable of driving rtCPA in naive animals. Consistent with previous studies (Do-Monte et al., 2017; Zhu et al., 2016), activation of the PVT→NAc pathway induced rtCPA in naive animals (Figure S1A; t_(10)_ = 2.434, p = 0.0352). To address any potential effects of light/heat in driving aversion during the rtCPA test, a separate cohort of naive animals underwent surgical implantation of the optic fiber in the PVT, but it did not receive ChR2, and was tested for rtCPA. Stimulation of the PVT (470 nm, 20 Hz) did not induce rtCPA in the absence of ChR2 (Figure S1B; t_(7)_ = 0.7361, p = 0.4856), indicating that light/heat effects are negligible under these stimulation conditions. Based on these results, we next investigated whether driving PVT→NAc-mediated aversion could also drive heroin seeking. Rats underwent heroin self-administration for 12 days (Figure S2A), followed by home cage abstinence, and then underwent a cued relapse test on withdrawal day 14 (i.e., cues, no drug; Figure 2A). As hypothesized, optogenetic stimulation (470 nm, 20 Hz) of the PVT→NAc pathway was sufficient to induce rtCPA after abstinence (Figure 2B; t_(5)_ = 3.411, p = 0.0190) and potentiated heroin seeking during the cued relapse test (Figure 2C; two-way repeated measures [RM] ANOVA, stim: F_(1,5)_ = 4,296 p = 0.0937; lever: F_(1,5)_ = 6.821, p = 0.0476; stim by lever interaction: F_(1,5)_ = 9.196, p = 0.0290; stim versus no stim: active lever: p = 0.0185; inactive lever: p = 0.9813), indicating that activation of the PVT→NAc pathway is sufficient to drive both aversion and relapse after protracted home-cage abstinence.

### Extinction blunts the ability of the PVT→NAc pathway to drive aversion and heroin relapse

**Figure 3.**
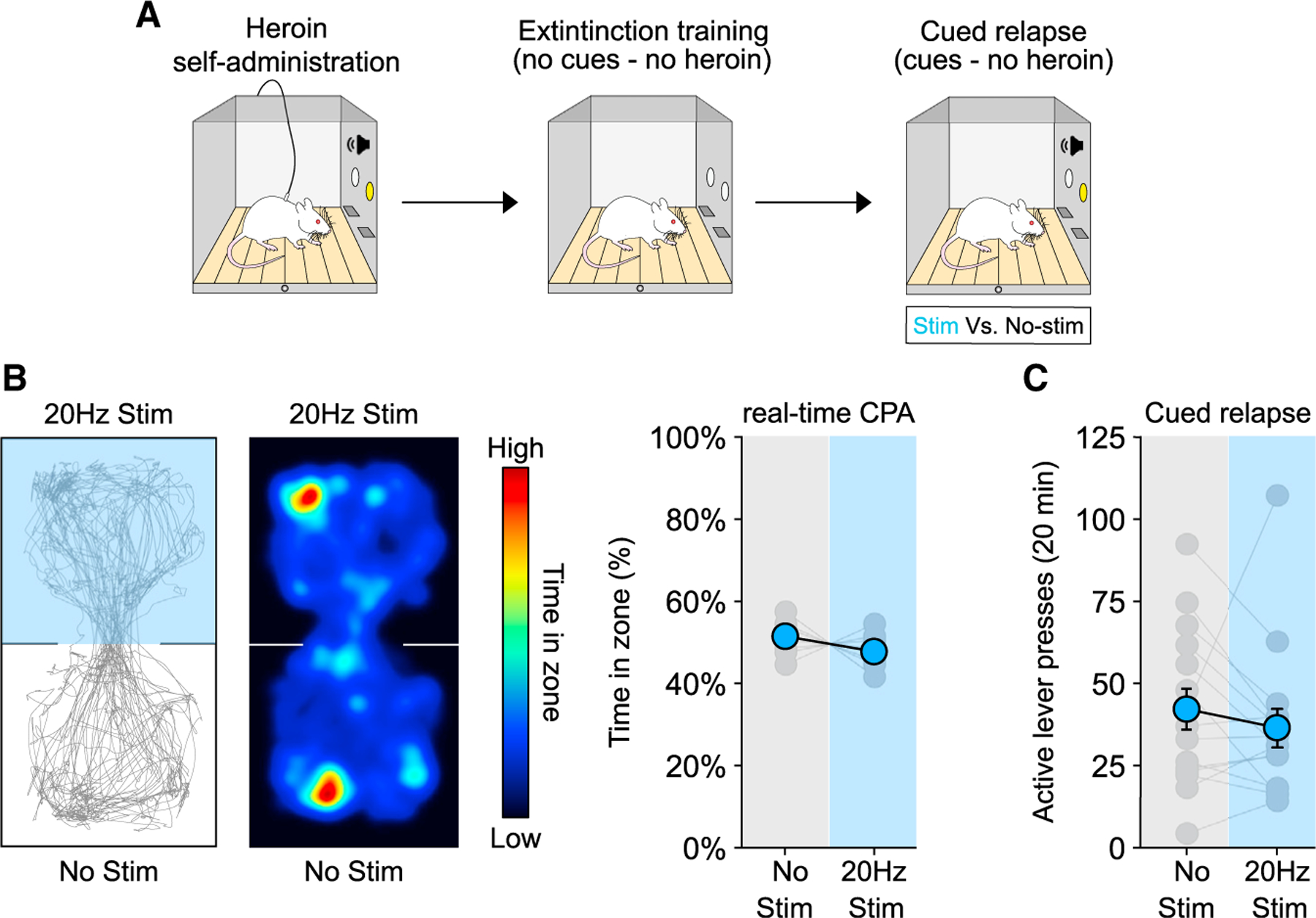
Extinction training blunts the ability of the PVT→NAc pathway to drive aversion and heroin relapse (A) Timeline employed for the behavioral experiments. (B and C) Example trace (left) and heatmap (middle) of real-time CPA test after extinction training from heroin self-administration (B). Optogenetic activation of the PVT→NAc pathway did not induce aversion (B, right; n = 9) and did not potentiate heroin relapse (C; n = 15) after extinction. Data are shown as mean ± SEM.

We next examined whether the ability of the PVT→NAc pathway to drive aversion and heroin seeking is similar after extinction training (Figure 3A). After 12 days of heroin self-administration (Figure S2B, left), rats underwent daily extinction training sessions (1-h duration) (Figure S2B, right; last 3 days) conducted without the heroin-associated (tone + light) cues until heroin seeking levels were low (9–14 days). Surprisingly, optogenetic activation of the PVT→NAc pathway after extinction training did not drive aversion (Figure 3B; t_(8)_ = 1.424, p = 0.1923). Consistent with this observation, PVT→NAc activation did not potentiate cued relapse after extinction (Figure 3C; two-way RM ANOVA, stim: F_(1,14)_ = 2.211 p = 0.1592; lever: F_(1,14)_ = 12.04, p = 0.0037; stim by lever interaction: F_(1,14)_ = 0.0098, p = 0.9224). Interestingly, stimulation of the PVT→NAc pathway during short-term (24 h) withdrawal from heroin self-administration was still capable of driving aversion (Figure S3B; t_(8)_ = 2.505, p = 0.0366) and heroin seeking on the first extinction session (i.e., no heroin, no cues) (Figure S3C; two-way RM ANOVA: stim: F_(1,14)_ = 3.558, p = 0.0802; lever: F_(1,14)_ = 41.17, p < 0.0001; stim by lever interaction: F_(1,14)_ = 5.100, p = 0.0404; stim versus no stim: active lever: p = 0.0126; inactive lever: p = 0.9999). These results indicate that extinction training blunts the ability of the PVT→NAc pathway to drive both aversion and relapse.

### Extinction alters synaptic plasticity in the PVT→NAc pathway

**Figure 4.**
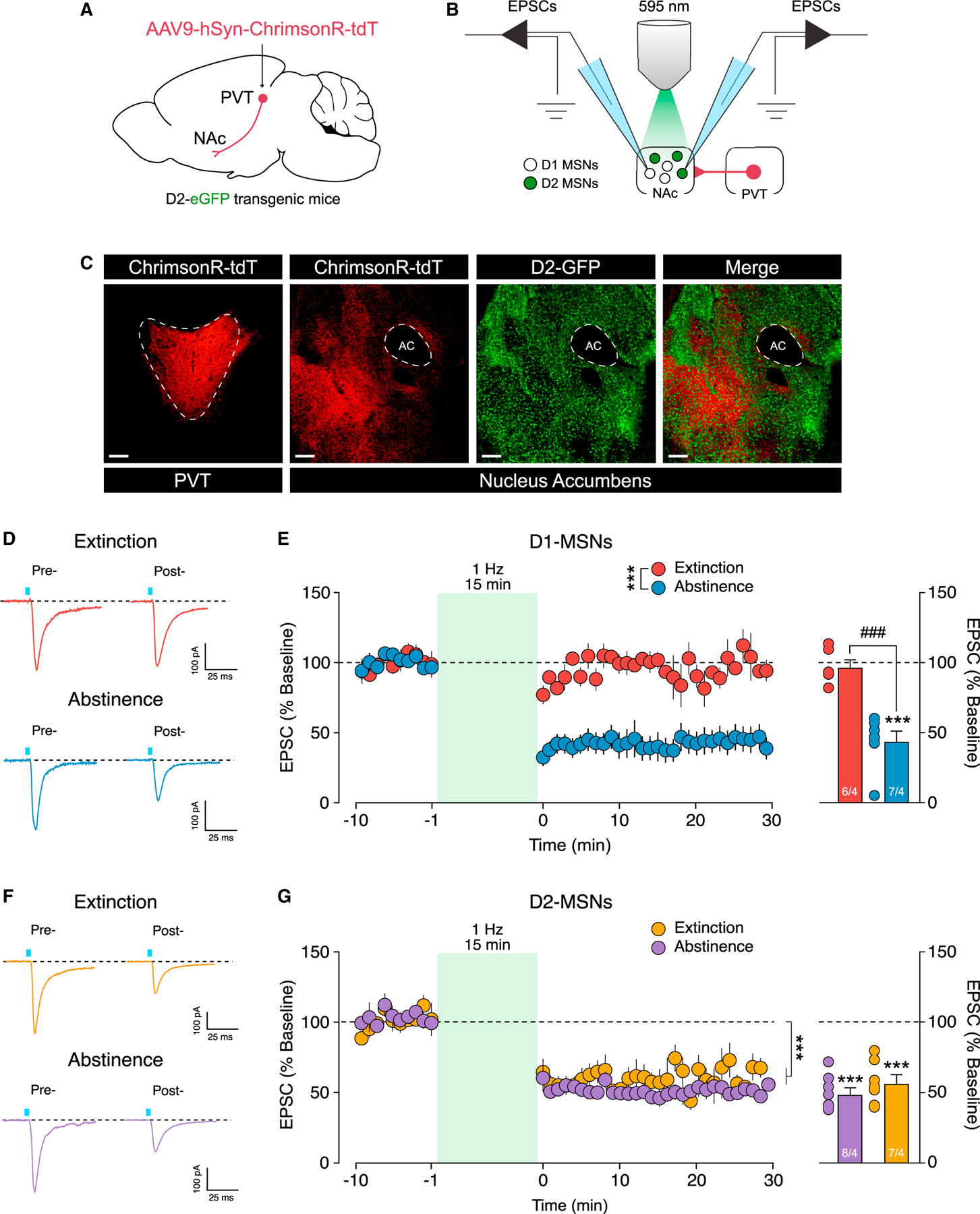
Extinction training induces a loss of synaptic plasticity in NAc D1-MSNs (A and B) Schematic illustration of the viral approach used in the D2-eGFP transgenic mice (A) and slide electrophysiology setup (B) employed in our study. (C) Representative image of ChrimsonR-tdT expression in the PVT (left, red), ChrimsonR-expressing terminals in the NAc (middle, red), eGFP expression in D2-NAc MSNs (middle, green), and overlap (green + red, right). (D) Example traces of EPSCs recorded in NAc D1-MSNs before (pre-, left) and after (post-, right) LTD protocol application following extinction (top) or abstinence (bottom). (E) Time course (left) of optically evoked EPSCs in D1-MSNs before and after LTD protocol application (***p < 0.001, group by time interaction). Extinction, but not abstinence, produces a loss of LTD in D1-MSNs (right) (extinction, n = 6 cells/4 rats; abstinence, n = 7 cells/4 rats; ***p < 0.001, baseline versus the average of the last 10 min post-LTD; ^###^p < 0.001, abstinence versus extinction). (F) Example traces of EPSCs recorded in NAc D2-MSNs before (pre-, left) and after (post-, right) LTD protocol application following extinction training (top) or abstinence (bottom). (G) Time course (left) of optically evoked EPSCs in D2-MSNs before and after LTD protocol application (***p < 0.001, main effect of time). Stimulation (1 Hz, 900 pulses) of ChrimsonR-expressing PVT terminals in the NAc drives LTD in D2-MSNs after abstinence and extinction (right) (extinction, n = 8 cells/4 rats; abstinence, n = 7 cells/4 rats; ***p < 0.001, baseline versus the average of the last 10 min post-LTD). Scale bars, 50 μm. Data are shown as mean ± SEM.

To determine the synaptic mechanisms by which extinction training alters the contribution of the PVT→NAc pathway to drive aversion and heroin seeking, we investigated the ability to induce synaptic plasticity in NAc MSNs after abstinence or extinction from heroin self-administration (Figure S2C, abstinence group; Figure S2D, extinction group). We first measured optogenetically evoked field excitatory postsynaptic potentials (fEPSPs) in the NAc before and after application of an LTD protocol (450 nm, 1 Hz, 900 pulses). This effectively induced LTD in the NAc fEPSPs after both abstinence and extinction, with no difference between groups (Figure S4A; two-way RM ANOVA: time: F_(79,790)_ = 11.24, p < 0.0001; group: F_(1,10)_ = 0.9643, p = 0.3493; time by group interaction: F_(79,790)_ = 0.7835, p = 0.9143). Importantly, note that the NAc contains a heterogeneous population of neurons. Most (~95%) belong to one of two types of MSNs, that is, those expressing the dopamine receptor 1 (D1-MSNs) or dopamine receptor 2 (D2-MSNs). Moreover, these distinct MSN subpopulations have opposing effects on drug seeking (Hearing et al., 2018; Heinsbroek et al., 2017; Lobo and Nestler, 2011; Pascoli et al., 2014) that might mask synaptic adaptations when plasticity is measured in the entire NAc population indiscriminately. Therefore, we employed D2-eGFP transgenic mice to investigate synaptic plasticity specifically in D1-(GFP^−^) versus D2-(GFP^+^) MSNs. The PVT was injected with ChrimsonR (Figure 4A) and mice underwent heroin self-administration for 12 days followed by extinction training or abstinence (Figure S2E, abstinence group; Figure S2F, extinction group). Coronal slices containing the NAc were obtained at withdrawal day 14, and EPSCs in D1- and D2-MSNs were recorded (Figure 4B) before and after application of the LTD protocol (595 nm, 1 Hz, 900 pulses). ChrimsonR-tdT expression was confined to the PVT and terminals were abundant in the NAc (Figure 4C). EPSCs were recorded in putative D1-MSNs (GFP^−^) before and after application of the LTD protocol in slices from extinction versus abstinence groups (Figure 4D). Interestingly, LTD was effectively induced in D1-MSNs from mice after abstinence, but not after extinction (Figure 4E, left: two-way RM ANOVA, time: F_(39,417)_ = 15.03 p < 0.0001; group: F_(1,11)_ = 47.45, p < 0.0001; time by group interaction: F_(39,417)_ = 10.84, p < 0.0001). Application of the LTD protocol significantly reduced the amplitude of the photo-evoked EPSCs after abstinence (Figure 4E, right; abstinence pre- versus post-LTD: t_(6)_ = 8.023, p = 0.0002), but not extinction (Figure 4E, right: extinction pre- versus post-LTD: t_(5)_ = 0.5819, p = 0.5859), compared to baseline, with a significant difference between the abstinence and extinction groups (Figure 4E, right: abstinence post-LTD versus extinction post-LTD: t_(11)_ = 6.017, p < 0.0001). This difference was only evident in D1-MSNs, as application of the same LTD protocol was able to drive LTD in D2-MSNs in both abstinence and extinction groups (Figures 4F and 4G; two-way RM ANOVA, time: F_(39,492)_ = 29.46, p < 0.0001; group: F_(1,13)_ = 1.687, p = 0.2166; time by group interaction: F_(39,492)_ = 0.6989, p = 9159; extinction pre- versus post-LTD: t_(7)_ = 11.76, p < 0.0001; abstinence pre- versus post-LTD: t_(6)_ = 7.489, p = 0.0003).

### Chemogenetic inhibition of the PVT→NAc pathway reduces heroin relapse after abstinence, but not extinction

**Figure 5.**
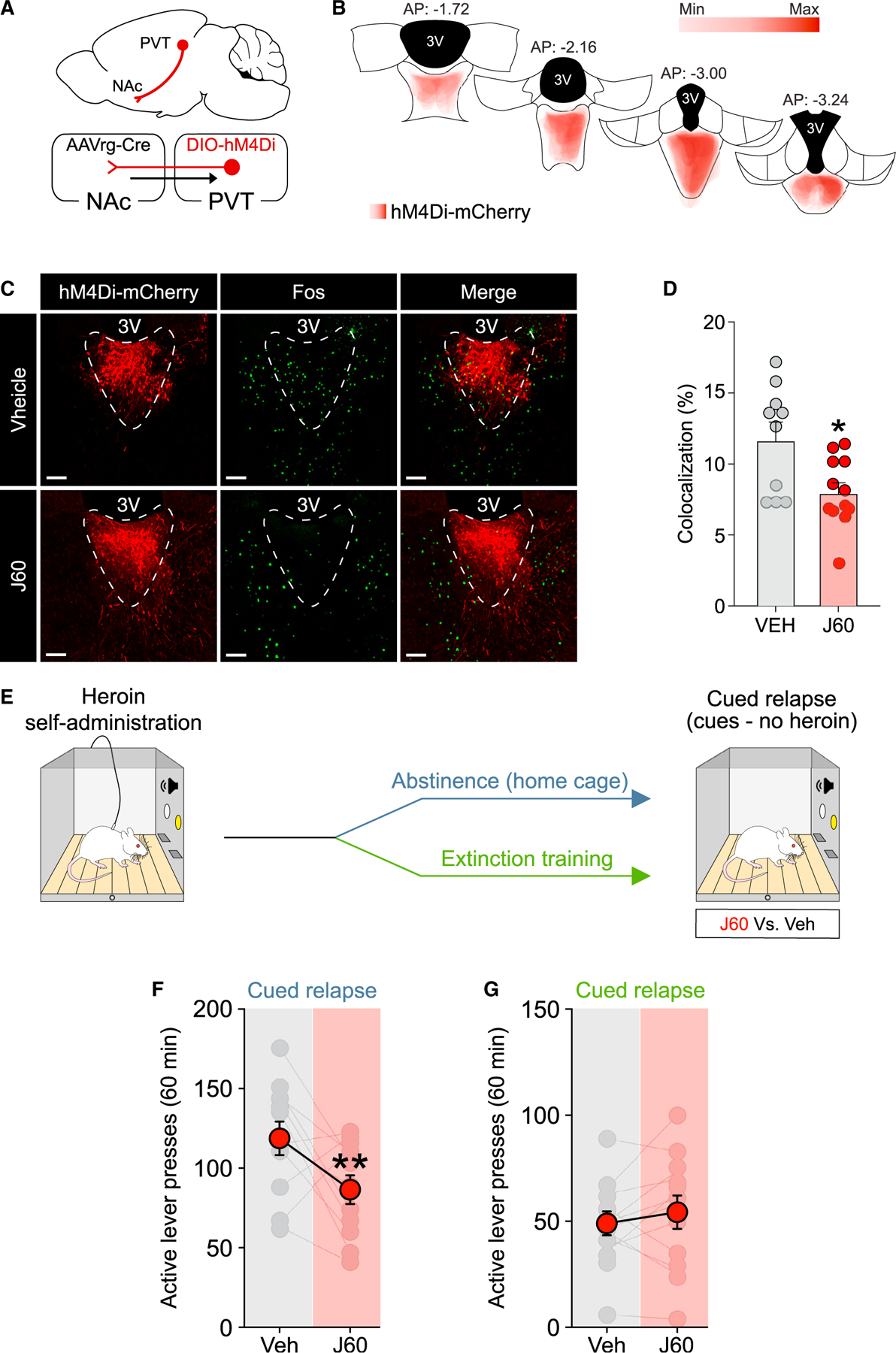
Chemogenetic inhibition of the PVT→NAc pathway reduces heroin relapse (A) Schematic illustration of the combinatorial virus approach employed in our study. (B) Placement map showing hM4Di-mCherry expression in the PVT (each animal represented at 8% opacity). (C and D) Chemogenetic inhibition of the PVT→NAc pathway with the DREADD agonist J60 reduced cocaine-induced Fos expression in hM4Di-mCherry-expressing neurons (vehicle [Veh], n = 10; J60, n = 12). (E) Timeline employed for the behavioral experiment. (F and G) Chemogenetic inhibition of the PVT→NAc pathway reduces heroin relapse after abstinence (F; n = 11) but not extinction training (G; n = 12) from heroin self-administration. *p < 0.05, **p < 0.01 versus vehicle. Scale bars, 50 μm. Data are shown as mean ± SEM.

One potential interpretation of the loss of LTD in PVT→NAc D1-MSNs after extinction is that these synapses may already be depressed by extinction training itself, occluding further LTD induction. If so, it should be possible to mimic this putative adaptation by acutely reducing activity in the PVT→NAc pathway. Thus, we hypothesized that chemogenetic inhibition of the PVT→NAc pathway would be able to reduce heroin relapse after abstinence, but not extinction. We used a retrograde AAVrg-Cre in the NAc and a Cre-dependent DIO-hM4Di in the PVT to restrict expression of the inhibitory (hM4Di) designer receptor exclusively activated by designer drug (DREADD) to the PVT→NAc pathway (Figure 5A) and verified that DREADD expression was restricted to the PVT (Figure 5B). In our chemogenetic experiments we used the new DREADD ligand JHU37160 (J60), which has been shown to have high selectivity for DREADDs and high *in vivo* DREADD affinity/potency at much lower doses than clozapine-*n*-oxide (CNO) (Bonaventura et al., 2019). In our experiments, we used a relatively low dose of J60 (0.1 mg/kg), which has been shown to bind approximately 80% of cortical hM4Di-DREADD in rats. Moreover, no off-target effects (measured as locomotion) and no changes in brain metabolic activity were found when J60 was administered to naive animals at the dose of 0.1 mg/kg (i.e., the dose used in our chemogenetic experiments) (Bonaventura et al., 2019). More importantly, to date, no off-target effects of J60 have been reported. The efficacy of the DREADD ligand J60 to reduce neuronal activity in animals expressing the inhibitory Gi-DREADD was verified by Fos analyses. J60 significantly reduced Fos expression induced by a priming injection of cocaine in DREADD^+^ neurons in the PVT compared to the vehicle group (Figures 5C and 5D) (vehicle versus J60: t_(20)_ = 2.8, p = 0.011).

Rats were trained to self-administer heroin followed by abstinence or extinction training (Figure S2G, abstinence group; Figure S2H, extinction group) and underwent a cued relapse test at withdrawal day 14 (Figure 5E). Consistent with the hypothesis, chemogenetic inhibition of the PVT→NAc pathway reduced heroin seeking during the cued relapse test after abstinence (Figure 5F, two-way RM ANOVA; treatment: F_(1,10)_ = 5.206, p = 0.0457; lever: F_(1,10)_ = 138.3, p < 0.0001; treatment by lever interaction: F_(1,10)_ = 5.226, p = 0.0453; vehicle versus J60: active lever: p = 0.0095, inactive lever: p = 0.9181). After extinction training, however, chemogenetic inhibition of the PVT→NAc pathway did not alter relapse rates during this test (Figure 5F, two-way RM ANOVA; treatment: F_(1,11)_ = 0.7457, p = 0.4063; lever: F_(1,11)_ = 54.73, p < 0.0001; treatment by lever interaction: F_(1,11)_ = 0.1072, p = 0.7495). These data indicate that the PVT→NAc pathway is necessary for heroin relapse after abstinence, but not after extinction, suggesting that inhibition of this pathway may simulate extinction-induced synaptic plasticity to reduce relapse propensity during protracted opioid withdrawal.

## DISCUSSION

In this study, we employed optogenetics and chemogenetics to investigate the role of the PVT→NAc pathway in aversion and two different forms of relapse to heroin seeking (e.g., abstinence versus extinction). We found that the PVT→NAc pathway is both sufficient and necessary to drive heroin seeking after abstinence, but not after extinction training. Our findings support previous studies indicating that the PVT→NAc pathway drives aversion and retrieval of opioid-associated memories after *non-contingent* morphine (Keyes et al., 2020; Zhu et al., 2016). Our results extend the aforementioned findings to contingent, self-administered heroin and, more importantly, reveal that extinction diminishes the ability of the PVT→NAc pathway to drive aversion and heroin relapse by altering synaptic plasticity within a specific subpopulation of NAc MSNs (D1-MSNs).

The most widely used animal models of drug addiction are the conditioned place preference (CPP) and drug self-administration models. The CPP test has been used primarily to assess the rewarding effects of drugs (Bardo and Bevins, 2000; Tzschentke, 2007), whereas self-administration models, wherein animals are allowed to regulate their own intake of a drug, are the gold standard in the field of preclinical addiction research (Reiner et al., 2019; Venniro et al., 2016). Self-administration models are used to measure drug-taking and drug-seeking behavior (reinforcing properties) (Bossert et al., 2013), and therefore have greater face validity to the human condition (Epstein et al., 2006; Spanagel, 2017). Although *non-contingent* drug administration has provided insight on how repeated drug exposure alters neuronal function, neuroplastic changes associated with *non-contingent* drug exposure do not always overlap with those mediated by *contingent* drug taking (Steketee and Kalivas, 2011).

In preclinical studies of drug self-administration, the drug withdrawal phase is typically characterized by an abstinent period in the home-cage or extinction training (Reiner et al., 2019; Venniro et al., 2016). Extinction of the drug-reinforced responding occurs in the absence of discrete cues (tone and light) previously paired with drug infusions during the training phase, and it results in reduced drug seeking over time. Abstinence, in contrast, involves no behavioral training but is often associated with a time-dependent increase in drug seeking, a phenomenon known as incubation of drug craving (Pickens et al., 2011). Relapse in both models is measured by lever pressing when the animals are returned to the behavioral chamber and allowed to respond for contingent presentations of the discrete cues (tone and light) previously paired with drug infusion (Reiner et al., 2019). Relapse after extinction versus abstinence depends on distinct neural circuitry (Farrell et al., 2018; Fuchs et al., 2006), with the former relying on prefrontal circuits and the latter on central amygdala circuits (Peters et al., 2009; Roura-Martínez et al., 2020; Venniro et al., 2017).

Based on emerging evidence in recent years, the PVT has been conceptualized to act as an emotional and motivational hub (Choi et al., 2019; Choi and McNally, 2017; Hsu et al., 2014; Kirouac, 2015). The PVT contributes to conditioned fear (Do-Monte et al., 2015; Penzo et al., 2015), conditioned sucrose (Do-Monte et al., 2017; Labouèbe et al., 2016; Livneh et al., 2017), appetitive learning (Otis et al., 2017), as well as drug seeking (Giannotti et al., 2018; Hamlin et al., 2009; James et al., 2010; Kuhn et al., 2018; Matzeu and Martin-Fardon, 2018; Zhou et al., 2021) and food seeking (Christoffel et al., 2021; Engelke et al., 2021), depending on specific PVT inputs and outputs (Millan et al., 2017; Penzo and Gao, 2021). Moreover, PVT neurons encode multiple salient features of sensory stimuli, including aversive valence (Li et al., 2011; Yasoshima et al., 2007; Zhu et al., 2018). In addition, PVT neurons increase their firing rate and exhibit an increased AMPA/NMDA (2-amino-3-(3-hydroxy-5-methyl-isoxazol-4-yl)propanoic acid/N-methyl-d-aspartate) current ratio 24 h after non-contingent morphine exposure in mice (McDevitt and Graziane, 2019), indicating that the PVT is recruited during early opioid withdrawal to drive aversive states (Zhu et al., 2016). This is consistent with our data, showing that activation of the PVT→NAc pathway during early (24 h) withdrawal from heroin self-administration drives aversion (e.g., rtCPA) and potentiates heroin seeking.

Zhu et al. (2016) demonstrated that the PVT→NAc pathway contributes to the somatic symptoms of opioid withdrawal. In this elegant work, they made mice dependent on morphine using repeated *non-contingent* injections and found that either spontaneous or naloxone-precipitated withdrawal induced Fos expression in the PVT neurons projecting to NAc, supporting the notion that activity in this pathway mediates the aversive component of morphine withdrawal. Moreover, optogenetic and/or chemogenetic inhibition of the PVT→NAc pathway attenuates both spontaneous and naloxone-precipitated withdrawal signs (Zhu et al., 2016). Altogether, these data suggest that activity of the PVT→NAc pathway is required for the aversive experience of opioid withdrawal.

Since optogenetic activation of the PVT→NAc is sufficient to drive a rtCPA in naive animals (Do-Monte et al., 2017; Zhu et al., 2016), we hypothesized that optogenetic activation of the PVT→NAc pathway would potentiate the aversive state experienced during heroin withdrawal and drive heroin seeking. We found that optogenetic activation of the PVT→NAc is sufficient to drive both aversion and heroin seeking after acute (24 h) and protracted (14 day) heroin withdrawal. At both time points, the same stimulation parameters were capable of potentiating heroin seeking, suggesting that precipitating an aversive state, such as that experienced during opioid withdrawal, contributes to relapse. After extinction, however, activation of the PVT→NAc was no longer able to drive aversion or relapse, indicating that extinction fundamentally alters the function of this pathway.

No studies to date have directly investigated the contribution of the PVT→NAc pathway to heroin seeking after abstinence versus extinction training. Our finding that the PVT→NAc pathway is both sufficient and necessary to drive heroin seeking after abstinence but not extinction points to an extinction-induced adaptation that alters the function of this pathway. Recently, Keyes et al. (2020) found that the PVT→NAc is necessary for the retrieval and maintenance of morphine memories in a CPP model. In this study, the authors showed that chemogenetic and optogenetic inhibition of PVT terminals in the NAc blocked retrieval, but not acquisition, of morphine CPP and conferred long-lasting protection against morphine-primed reinstatement, leading the authors to speculate that inhibition of the PVT→NAc pathway may deepen extinction of morphine memories (Keyes et al., 2020). In our study, chemogenetic inhibition of this pathway reduced relapse after abstinence, but not extinction, and, interestingly, the relapse rates in abstinent rats were constrained to levels observed after extinction training. These data are consistent with the notion that inhibition of the PVT→NAc pathway may simulate the neuroplastic effects of extinction training, thus precluding further reductions in relapse after extinction.

Keyes et al. (2020) also demonstrated that short-term withdrawal (2 days) from repeated non-contingent morphine injections potentiates PVT synapses onto D2-MSNs. They suggested that the excitatory PVT input to these GABAergic D2-MSNs increases lateral inhibition of D1-MSNs projecting to the lateral hypothalamus, leading to retrieval of morphine-associated memories and relapse (Keyes et al., 2020). Our findings, in contrast, indirectly suggest that at protracted withdrawal time points (14 days), PVT synapses onto D1-MSNs may be depotentiated after extinction (but not abstinence) from self-administered heroin. The complexity of these MSN subpopulations is underscored by observations that even within a given subpopulation, differential projections can elicit opposite effects on drug seeking (Gibson et al., 2018). Our results are nonetheless consistent with the notion that D1-MSNs are generally thought to drive drug seeking, whereas D2-MSNs exert an opposing inhibitory action on drug seeking (Hearing et al., 2018; Heinsbroek et al., 2017; Lobo and Nestler, 2011; Pascoli et al., 2014). Our finding that LTD is lost specifically in D1-MSNs after extinction, but not abstinence, suggests that PVT→NAc inputs to D1-MSNs may have been depotentiated by extinction training itself. This is one potential mechanism whereby extinction-induced synaptic plasticity could reduce heroin seeking, by diminishing the aversion signal in the PVT→NAc pathway that promotes relapse. This could also explain why chemogenetic inhibition of the PVT→NAc pathway was able to reduce heroin relapse only after abstinence (but not extinction), and why the effect size was similar to the reduction in relapse achieved by extinction training.

Overall, these results point to the PVT→NAc pathway as a key component of the neural circuitry driving aversion and heroin relapse after abstinence and identify this pathway as a potential substrate by which extinction training reduces relapse. Moving forward, it will be necessary to identify whether specific inputs to PVT regulate its ability to drive aversion and relapse through its output to the NAc. Finally, our data suggest that treatment strategies that emulate extinction-induced synaptic plasticity may reduce relapse rates, and identify the PVT→NAc pathway as a potential point of intervention to alleviate aversion during opioid withdrawal and reduce opioid craving.

## STAR★METHODS

### RESOURCE AVAILABILITY

#### Lead contact

Further information and requests for resources and reagents should be directed to and will be fulfilled by the lead contact, Jamie Peters (jamie.l.peters@cuanschutz.edu).

#### Materials availability

This study did not generate new unique reagents.

#### Data and code availability

- Data reported in this paper will be shared by the lead contact upon request.
- This study did not generate unique code.
- Any additional information required to reanalyze the data reported in this paper is available from the Lead Contact upon request.

### EXPERIMENTAL MODEL AND SUBJECT DETAILS

Our experimental procedures followed the guidelines outlined in the *Guide for the Care and Use of Laboratory Animals* (National Research Council (US) Committee for the Update of the Guide for the Care and Use of Laboratory Animals, 2011) and conducted in a AAALAC accredited facility.

#### Rats

Subjects were age-matched (P55–60) single housed male and female Wistar rats (Charles River; Raleigh, NC). Animals were housed in a temperature and humidity-controlled environment with a 12 h light/dark cycle (8:00 A.M. lights on) with free access to standard laboratory chow and water. Rats (n = 13) were excluded from the final dataset due to defective optogenetic implants (n = 6) or poor virus expression (n = 9). All animal procedures were approved by the Medical University of South Carolina and the University of Colorado-Denver, Anschutz Medical Campus Institutional Animal Care and Use Committee (IACUC).

#### Mice

Subjects were adult male and female D2-eGFP transgenic mice between 30 to 40 weeks of age (MMRRC stock S118Gsat/Mmnc; obtained from Dr. Peter Kalivas). Animals were grouped housed in a temperature and humidity-controlled environment with a 12 h light/dark cycle (8:00 A.M. lights on) with free access to standard laboratory chow and water. One mouse was excluded from the final dataset due to poor virus expression. Mice were bred and maintained according to protocols approved by the University of Colorado-Denver, Anschutz Medical Campus Institutional Animal Care and Use Committee (IACUC).

### METHOD DETAILS

#### Intravenous surgery

Rats were anesthetized with ketamine/xylazine (80/7 mg/kg) and implanted with an intravenous catheter. The distal end of the silastic tubing was inserted into the right jugular vein; the other end was passed subcutaneously over the shoulder to a 22-gauge cannula mounted on the animal’s back. Ketamine boosters were given as needed to maintain the surgical plane of anesthesia. Ketorolac (15 mg/kg) or carprofen (5 mg/kg) and cefazolin (30–200 mg/kg) were administered to alleviate surgical pain and prevent infection, respectively. To prevent catheter occlusion, the catheter locking solution, taurolidine-citrate (TCS), was used. Rats were allowed to recover from surgery at least 1 week before heroin self-administration training. Intravenous surgeries in mice were performed as described previously (Heinsbroek et al., 2017). In brief, mice were anesthetized with isoflurane (induction 3%–5% v/v, maintenance 1%–2% v/v) and implanted with an indwelling catheter inserted 11 mm into the right jugular vein, locked with TCS. Carprofen (5 mg/kg), cefazolin (200 mg/kg) and topical antibacterial ointment were given for 2–3 d following surgery. Afterward catheters were flushed daily with heparinized (100 units/ml) saline solution and cefazolin to maintain patency and prevent infection. Mice were single-housed following surgery and allowed to recover 7–10 d before heroin self-administration training.

#### Viral injections and optogenetic probe implantation

For optogenetic experiments in rats, the NAc was bilaterally injected with 1 μl of AAVrg-EF1a-mCherry-IRES-Flpo (8×10^12^ vg/ml) and the PVT was injected with 0.3 μl of AAVDj-Ef1a-fDIO-hChR2(H134R)-eYFP-WPRE (5.6×10^12^ vg/ml). For chemogenetic experiments in rats, the NAc was bilaterally injected with 1μl of AAVrg-pENN-AAV-hSyn-CRE-WPRE-hGH (5×10^12^ vg/ml) and the PVT was injected with 0.7 μL of AAV2-hSyn-DIO-hM4D(Gi)-mCherry (5×10^12^ vg/ml). All viral injections in the NAc were performed using the following coordinates according to Paxinos and Watson (2007) and relative to Bregma: anteroposterior (A/P) +1.6 mm; mediolateral (M/L) ± 0.8 mm; dorsoventral (D/V) −7.5 mm. Viral injections in the PVT were performed using the following coordinates: A/P −3 mm; M/L +2.42 mm (25° angle); D/V −5.74 mm. All viral injections were performed with a nanoliter injector Nanoject III (Drummond Scientific, Broomall, PA, USA) at the rate of 20 nl/s using a borosilicate glass tip. At the end of the infusion, the glass tip was left in place for an additional 10 min to allow diffusion of the virus. A 470 nm wireless optogenetic probe (Neurolux, Evanston, IL, USA; Shin et al., 2017) or fiber optic cannula (2.5 mm ferrule diameter, 200 μm core, 0.37 NA; ThorLabs, Newton, NJ, USA) was implanted in the PVT one week after virus infusion under isoflurane anesthesia (5% induction v/v; 2%–3% maintenance v/v) using the following coordinates relative to Bregma: A/P −2.6 mm; M/L +0.5 mm (5° angle); D/V −6 mm. Optogenetic implants were anchored to the skull using screws and dental cement.

#### Heroin self-administration

After recovery from intracranial surgery, rats underwent 12 days of heroin self-administration in standard operant chambers placed in ventilated, sound-attenuating cubicles (Med Associates, St. Albans, VT, USA). Rats were initially trained to self-administer heroin on a fixed-ratio 1 (FR1) schedule of reinforcement over daily sessions (3 h) at the dose of 0.04 mg/infusion. During the self-administration sessions, a light above the active (right) lever and 3.5 kHz tone are turned on (5 s) coincident with the onset of each heroin infusion (2.85 s – 50 μl). Responses on the inactive (left) lever were recorded but had no programmed consequences. Each rewarded lever press was followed by a 20 s time-out period, signaled by the house-light off, wherein heroin was unavailable. After 7 sessions on an FR1 schedule, training progressed to an FR2 (2 sessions) and ends on an FR4 (3 sessions). Diamorphine HCl (heroin; NIDA Drug Supply Program, Bethesda, MD) was dissolved in 0.9% sterile saline at the final concentration of 0.8 mg/ml. Heroin self-administration in mice was performed under the same experimental conditions described above. In brief, 7–10 days after surgery, mice were food-deprived overnight and trained to self-administer heroin (0.15 mg/kg/infusion) for 12 days under the same schedule of reinforcement described above. During heroin self-administration, operant responses (active nose pokes) for heroin were paired with the presentation of discrete cues (tone + light) for 3 s, followed by a 10 s time-out period wherein heroin was unavailable. Inactive nose pokes were recorded but had no programmed consequences. Catheter patency was verified as needed by infusing methohexital sodium, which induces a brief loss of righting reflex.

#### Acute withdrawal test and optogenetic stimulation

Twenty-four hours after the last heroin self-administration session, rats underwent a 20 min extinction test (i.e., the first extinction day - no-cues, no-heroin) conducted in a within-subject design. We chose this approach to account for individual differences over multiple testing time points (Tye and Deisseroth, 2012). The order of test conditions (stim versus no-stim) was randomized, such that half the animals received stim on the first test. In between tests, rats underwent 2 additional days of heroin self-administration (FR4). Stimulation (20 Hz, 20 ms) was delivered using the wireless probe (470 nm, Shin et al., 2017) or a 470 nm laser line (Shanghai Laser & Optics Century, Shanghai, China) connected to a patch cord (200 μm core, 0.37 NA, Doric Lenses, Québec, Canada). Laser output was measured using a power meter (PM160 wireless power meter, Thorlabs, Newton, NJ, USA) and adjusted to ~10 mW (steady state) at the fiber tip. Square waves (20 Hz, 20 ms) for optogenetic stimulation were generated using Med-State Notation custom code driving a 28 V to TTL adaptor (Med Associates, St. Albans, VT, USA) connected to the laser line via a BNC cable. For the real-time CPA test, optogenetic stimulation was delivered in real time using the wireless probe (470 nm) or a 470 nm laser line with a custom-made Bonsai workflow (Lopes et al., 2015) triggering an Arduino UNO microcontroller (square wave-form generator of 20 Hz, 20 ms) each time the rat entered in the designated stim compartment.

#### Cued relapse test

After the acute (24 h) withdrawal testing, rats underwent daily extinction training in 1-h sessions until reaching extinction criteria (animals required 7–10 extinction sessions over 9–14 withdrawal days to reach criteria of < 20 active lever presses for 3 consecutive sessions). Twenty-four hours after the last extinction day, animals underwent a 20 min cued relapse test, wherein active lever presses resulted in the presentation of heroin cues (light + tone on an FR2 schedule of reinforcement). The cued relapse test was conducted using a within-subjects design wherein the same rat underwent a test with (stim) and without (no-stim) optogenetic stimulation in a counterbalanced order, as with the acute withdrawal test. Optogenetic stimulation (470 nm, 10 mW – 20 Hz, 20 ms) was delivered for the entire session and between tests, rats underwent 2 additional days of extinction training to re-establish a low baseline of heroin seeking. A separate cohort of animals underwent heroin self-administration followed by 14 d of forced abstinence instead of extinction training. This cohort was then tested in a cued relapse test (i.e., cues, no heroin) using a within-subjects, within-session design. During the 40 min test, rats received stim or no-stim in 10-min blocks, in a counter-balanced order, wherein no two consecutive blocks were the same condition. For chemogenetic experiments, after 12 days of heroin self-administration, rats underwent either 1-h daily extinction training session for 7 d (total of 14 d of withdrawal) or home cage abstinence for 14 d. Rats were then tested in a 1-h cued relapse test under extinction conditions (i.e., cues, no heroin on an FR2 schedule of reinforcement) conducted in a within-subjects design, with the treatment order counter-balanced across subjects and testing days. The DREADD ligand JHU37160 dihydrochloride (J60, HelloBio), dissolved in sterile water, was administered at a dose of 0.1 mg/kg (1 ml/kg, IP) 15 min prior to testing (Bonaventura et al., 2019).

#### Real-time conditioned place aversion (rtCPA)

Rats were tested for real-time conditioned place aversion (rtCPA) in a custom-made black acrylic apparatus (60 cm long × 30 cm wide × 40 cm high) with two identical compartments for 20 min. During the rtCPA test, one side of the apparatus delivered optogenetic stimulation (470 nm, 10 mW – 20 Hz, 20 ms). Testing occurred at least one hour after extinction/relapse tests. Behavior during the rtCPA test was recorded using a 180-degree wide angle camera at 30 fps, located above the apparatus, and was analyzed using EthoVision XT (EthoVision XT) tracking software.

#### Fos immunohistochemistry

At the end of experiments, rats were randomly assigned to two different groups: one group (n = 11) received optogenetic stimulation (stim - 470nm, 10mW – 20 Hz, 20 ms) for 20 min while the control group (n = 8) did not receive any light stimulation (no-stim). To verify the Gi-DREADD functionality, rats received an injection of cocaine (cocaine hydrochloride, 10 mg/kg, 1 ml/kg IP, NIDA Drug Supply) to induce Fos, 15 min after J60 (0.1mg/ kg, 1 ml/kg, n = 12) or sterile water pretreatment (n = 10). Two hours later, rats were transcardially perfused with 0.9% saline followed by 10% formalin. Brains were extracted, post-fixed for 1 h in 10% formalin, cryoprotected in 30% sucrose for at least 48 h, and stored at −80° until sectioning. Serial coronal sections (40 μm) were obtained using a Leica cryostat (CM-3050S and CM-1950; Leica Microsystems Inc., Buffalo Grove, IL), and free-floating sections were collected in chilled 0.1M PBS buffer containing 1% Sodium Azide. Free-floating sections were incubated for 1 h in 2% normal donkey serum (NDS; Jackson ImmunoResearch Labs) in PBS with 0.3% Triton X-100 (PBS-T) and then incubated overnight at 4°C with rabbit anti-c-Fos (1:2000 in 2% NDS in PBS-T, Millipore), rabbit anti-ser32-phospho-c-Fos (1:2000 in 2% NDS in PBS-T, Cell Signaling) and chicken anti-mCherry primary antibodies (1:5000, LifeSpan). Sections were then rinsed in PBS-T (3 × 10 min) and incubated for 2 h in PBS-T with 2% of NDS containing donkey anti-Rabbit Alexa Fluor 680 (1:500, Jackson ImmunoResearch Labs), donkey anti-Chicken Alexa Fluor 594 secondary antibodies (1:500, Jackson ImmunoResearch Labs) or donkey anti-Chicken Alexa Fluor 488 secondary antibodies (1:500, Jackson ImmunoResearch Labs). Sections were mounted onto slides and coverslipped with Fluoromount-G (Electron Microscopy Sciences, Hatfield, PA) before confocal imaging.

#### Image acquisition and cell counting

Z stacks (~20 μm) were acquired with a confocal microscope (Leica SP5 or Olympus FV1200 – 10X air objective, 1024×1024 frame size, 12-bit resolution, 4 frame averages). Z stacks were then imported in Imaris software (Bitplane), automatic threshold was set, and the number of Fos^+^ neurons in the PVT was automatically estimated using the Imaris spot detection function. The number of Fos^+^ neurons was then averaged across four PVT sections for each rat.

#### Electrophysiological validation of the optogenetics strategy

A separate cohort of rats (n = 3) was used for electrophysiological validation of the optogenetic parameters. The NAc of rats was bilaterally injected with 1 μl of AAVrg-EF1a-mCherry-IRES-Flpo and the PVT of rats was injected with 1 μl of AAVDj-Ef1a-fDIO-hChR2(H134R)-eYFP-WPRE as previously described. Four weeks after virus injection, coronal rat brain slices containing the NAc (240 μm) or PVT (180 μm) were prepared using a vibratome (VT 1200S, Leica) in an ice-cold sucrose solution containing (in mM) 75 NaCl, 2.5 KCl, 6 MgCl_2_, 0.1 CaCl_2_, 1.2 NaH_2_PO_4_, 25 NaHCO_3_, 2.5 D-glucose and 50 sucrose. Slice were incubated at 32°C in oxygenated artificial cerebrospinal fluid (aCSF) containing (in mM) 126 NaCl, 2.5 KCl, 1.2 MgCl_2_, 2.5CaCl_2_, 1.2 NaH_2_PO_4_, 21.4 NaHCO_3_, 11.1 D-glucose for 1 h before recording. Slices were then transferred to the recording chamber and perfused with 2 mL/min aCSF (32 ± 2°C). NAc and PVT neurons were visualized using an upright BXWI51 microscope (Olympus) with an infrared gradient contrast optics. Whole-cell recordings were made in the PVT or NAc using an Axopatch 200B amplifier (Molecular Devices) and data were acquired with Axograph X (Axograph, RRID:) at 10 KHz and filtered to 2 KHz for voltage-clamp recordings. Patch pipettes (1.5 – 2 MΩ, World Precision Instruments) for whole cell recordings of ChR2 currents and action potentials in PVT neurons contained (in mM): 135 KCl, 0.1 CaCl_2_, 2 MgCl_2_, 10 HEPES(K), 0.1 EGTA, 1 mg/mL ATP, 0.1 mg/mL GTP, and 1.5 mg/mL sodium phosphocreatine (pH = 7.3, 275 mOsm). To examine potential synaptic events driven by optogenetic stimulation of PVT terminals in the NAc, patch pipettes were filled with internal solution containing (in mM): 135 D-gluconic acid (K), 10 HEPES (K), 0.1 CaCl_2_, 2 MgCl_2_, 0.1 EGTA, 1 mg/mL ATP, 0.1 mg/mL GTP, and 1.5 mg/mL sodium phosphor-creatine (pH = 7.3, 275 mOsm). Neurons were held at a voltage of −60 mV. Optogenetic stimulation of PVT terminals in NAc was delivered using wide-field illumination (pulse width = 3 ms) through a blue LED (470 nm - Thorlabs, Newton, NJ, USA). All drugs were applied via perfusion.

#### Long-term depression (LTD) in the PVT→NAc pathway *ex vivo*

For LTD experiments, the PVT of the D2-eGFP transgenic mice (n = 9) was injected with 0.2 μl of AAV9-Syn-ChrimsonR-tdT (1×10^13^ vg/ml) using the following coordinates according to Bregma: A/P: −1.3; M/L: 0.5 (10° angle); D/V: −3.1. After abstinence or extinction from heroin self-administration (at 14 d of withdrawal) coronal slices containing the NAc (240 μm) were prepared using a vibratome (VT 1200S, Leica) in an ice-cold sucrose solution containing (in mM) 75 NaCl, 2.5 KCl, 6 MgCl_2_, 0.1 CaCl_2_, 1.2 NaH_2_PO_4_, 25 NaHCO_3_, 2.5 D-glucose and 50 sucrose. Slice were incubated at 32°C in oxygenated artificial cerebrospinal fluid (ACSF) containing (in mM) 126 NaCl, 2.5 KCl, 1.2 MgCl_2_, 2.5CaCl_2_, 1.2 NaH_2_PO_4_, 21.4 NaHCO_3_, 11.1 D-glucose for 1 h before recording. Slices were then transferred to the recording chamber and perfused with 2 mL/min ACSF (32 ± 2°C). Patch pipettes for whole cell recordings were filled with internal solution containing (in mM): 135 D-gluconic acid (K), 10 HEPES (K), 0.1 CaCl_2_, 2 MgCl_2_, 0.1 EGTA, 1 mg/mL ATP, 0.1 mg/mL GTP, and 1.5 mg/mL sodium phosphor-creatine (pH = 7.3, 275 mOsm). Neurons were held at a voltage of −60 mV and all recordings were acquired with Axograph X (Axograph, RRID:SCR_014284) at 10kHz. The D2-MSNs were identified by visualizing the eGFP tag using an upright BXWI51 microscope (Olympus) under dim light illumination (470 nm). Excitation of ChrismonR-expressing PVT terminals in the NAc was performed with a 595 nm-emitting LED (pulse width = 3 ms, Thorlabs). Optically evoked EPSCs in putative D1(eGFP−) and D2(eGFP+) MSNs were recorded before (10 min, baseline) and after (30 min) LTD protocol application (595 nm – 1 Hz, 4 ms – 900 pulses, 15 min) (Zhu et al., 2016). No series resistance compensation was used, and neurons were discarded if series resistance was over 15 MΩ. For LTD experiments in extracellular field recordings, the PVT of rats (n = 7) was injected with 0.75 μl of AAV9-hSyn-hChR2(H134R)-eYFP (5×10^12^ vg/ml) as previously described and rats were trained to self-administer heroin for 12 d. Following extinction training or abstinence (at 14 d of withdrawal), rats were transcardially perfused with ice-cold aCSF and coronal rat brain slices containing the NAc (300–400 μm) were prepared using a vibratome (VT 1200S, Leica), incubated at room temperature in oxygenated aCSF for 1-h before recording. Slices were transferred to a recording chamber continuously perfused with aCSF (1.5 ml/min at 31°C). Optogenetic-evoked extracellular field excitatory postsynaptic potentials (fEPSPs) were recorded by stimulating ChR2-expressing PVT terminals in the NAc with a custom made 450 nm laser line coupled to a patch cord (200 μm core, 0.37 NA, Doric Lenses, Québec, Canada). The tip of the patch cord was positioned directly above the slice (30° angle) ~400 μm from the recording electrode filled with 3 M NaCl solution. Photo-evoked fEPSPs were recorded every 20 s (450 nm – 15 mW, 4 ms) before (20 min, baseline) and after (60 min) LTD protocol application (1 Hz, 4 ms – 900 pulses, 15 min). Data were collected with the DataWave software (SciWorks) and the slope was extracted by calculating the derivative of the fEPSPs. Time series were obtained by compressing the slope of the fEPSP to 1-minute average and normalized as percentage of baseline.

### QUANTIFICATION AND STATISTICAL ANALYSIS

All statistical tests were performed in Prism (GraphPad Prism) statistical software. Statistical significance is denoted in the figures as *p < 0.05, **p < 0.01, ***p < 0.001 and the exact p values are reported in the Results. Biological replicates, denoted as n values, are listed in the figure legends for each experiment. The rtCPA data were analyzed using a two-tailed paired t test. Reinstatement tests (within-subject design) were analyzed using a two-way repeated-measures (RM) analysis of variance (ANOVA) with the lever (active and inactive) and type of stimulation (stim and no-stim) or treatment (J60 and vehicle) as within-subject factors followed by Sidak post hoc tests. Time course for slice electrophysiology and extracellular field potential experiments were analyzed with a two-way RM ANOVA on normalized data (percentage of baseline), with time as within-subject factors and group (abstinence and extinction) as between-subject factor. A two-tailed paired t test was used to compare the baseline (100%) with the average of the last 10 min of recordings after LTD protocol application. A two-tailed paired t test was used to compare the EPSCs amplitude before and after application of the AMPA receptor antagonist 6,7-dinitroquinoxaline-2,3-dione (DNQX). Fos data were analyzed with a two-tailed un-paired t test. Significance was defined as alpha < 0.05 and data are graphed as mean ± SEM.

## Supplementary Material

1

2

## Figures and Tables

**Table:** KEY RESOURCES TABLE

REAGENT or RESOURCE	SOURCE	IDENTIFIER
Antibodies		
rabbit anti-c-Fos	Millipore	RRID: AB_2631318
rabbit anti-ser32-phospho-c-Fos	Cell Signaling	RRID:AB_10557109
chicken anti-mCherry	LifeSpan	RRID: AB_2716246
donkey anti-Rabbit Alexa Fluor® 680	Jackson ImmunoResearch Labs	RRID:AB_2340627
donkey anti-Chicken Alexa Fluor® 594	Jackson ImmunoResearch Labs	RRID:AB_2340377
donkey anti-Rabbit Alexa Fluor® 488	Jackson ImmunoResearch Labs	RRID:AB_2313584
Bacterial and virus strains		
AAV9-hSyn-hChR2(H134R)-EYFP	Addgene	# 26973-AAV9
AAV2-hSyn-DIO-hM4D(Gi)-mCherry	Addgene	# 44362-AAV2
AAVrg-EF1a-mCherry-IRES-Flpo	Addgene	# 55634-AAVrg
AAVrg-pENN-AAV-hSyn-CRE-WPRE-hGH	Addgene	# 105553-AAVrg
AAV9-Syn-ChrimsonR-tdT	Addgene	#59171-AAV9
AAVDj-Ef1a-fDIO-hChR2(H134R)-eYFP-WPRE	UNC Vector Core	Fenno et al., 2014
Experimental models: Organisms/strains		
Drd2-eGFP transgenic mice # STOCK Tg(Drd2-EGFP)S118Gsat/Mmnc	Gong et al., 2003	RRID:MMRRC_000230-UNC
Wistar rats	Charles River	Strain code: 003
Software and algorithms		
Prism (V9.1.0)	GraphPad	RRID: SCR_002798
Python (V3.9.6)	Python software foundation	RRID:SCR_008394
Affinity Designer (V1.9.3)	Affinity	RRID: SCR_016952
Imaris (V9.0.1)	Bitplane	RRID: SCR_007370
Axograph X (V1.7.6)	Axograph	RRID:SCR_014284
DataWave (V7.2)	SciWorks	N/A
EthoVision XT (V14)	Noldus	RRID:SCR_000441
MedPC IV	Med Associates	RRID:SRC_012156
Other		
Normal donkey serum (NDS)	Jackson ImmunoResearch Labs	RRID:AB_2337258
JHU37160 dihydrochloride (J60)	HelloBio	# HB6261
Diamorphine HCl (Heroin)	NIDA Drug Supply Program	N/A
Cocaine hydrochloride	NIDA Drug Supply Program	N/A
6,7-dinitroquinoxaline-2,3-dione (DNQX)	Tocris	Cat #: 2312
Fluoromount-G	EM Science	RRID:AB_2572296

## Highlights

- The PVT→NAc pathway drives heroin relapse after abstinence
- Extinction training during withdrawal reduces relapse rates
- Extinction causes a loss of synaptic plasticity specifically in PVT→NAc D1-MSNs
- Extinction diminishes the ability of the PVT→NAc pathway to drive heroin relapse

## References

[R1] Bardo MT, and Bevins RA (2000). Conditioned place preference: what does it add to our preclinical understanding of drug reward? Psychopharmacology (Berl.) 153, 31–43. 11255927 10.1007/s002130000569

[R2] Bonaventura J, Eldridge MAG, Hu F, Gomez JL, Sanchez-Soto M, Abramyan AM, Lam S, Boehm MA, Ruiz C, Farrell MR, (2019). High-potency ligands for DREADD imaging and activation in rodents and monkeys. Nat. Commun 10, 4627. 31604917 10.1038/s41467-019-12236-z PMC6788984

[R3] Bossert JM, Marchant NJ, Calu DJ, and Shaham Y (2013). The reinstatement model of drug relapse: Recent neurobiological findings, emerging research topics, and translational research. Psychopharmacology (Berl.) 229, 453–476. 23685858 10.1007/s00213-013-3120-y PMC3770775

[R4] Choi EA, and McNally GP (2017). Paraventricular thalamus balances danger and reward. J. Neurosci 37, 3018–3029. 28193686 10.1523/JNEUROSCI.3320-16.2017 PMC6596734

[R5] Choi EA, Jean-Richard-Dit-Bressel P, Clifford CWG, and McNally GP (2019). Paraventricular thalamus controls behavior during motivational conflict. J. Neurosci 39, 4945–4958. 30979815 10.1523/JNEUROSCI.2480-18.2019 PMC6670259

[R6] Christoffel DJ, Walsh JJ, Heifets BD, Hoerbelt P, Neuner S, Sun G, Ravikumar VK, Wu H, Halpern CH, and Malenka RC (2021). Input-specific modulation of murine nucleus accumbens differentially regulates hedonic feeding. Nat. Commun 12, 2135. 33837200 10.1038/s41467-021-22430-7 PMC8035198

[R7] Do-Monte FH, Quiñones-Laracuente K, and Quirk GJ (2015). A temporal shift in the circuits mediating retrieval of fear memory. Nature 519, 460–463. 25600268 10.1038/nature14030 PMC4376623

[R8] Do-Monte FH, Minier-Toribio A, Quiñones-Laracuente K, Medina-Colón EM, and Quirk GJ (2017). Thalamic regulation of sucrose seeking during un-expected reward omission. Neuron 94, 388–400.e4. 28426970 10.1016/j.neuron.2017.03.036 PMC5484638

[R9] Dong X, Li S, and Kirouac GJ (2017). Collateralization of projections from the paraventricular nucleus of the thalamus to the nucleus accumbens, bed nucleus of the stria terminalis, and central nucleus of the amygdala. Brain Struct. Funct 222, 3927–3943. 28528379 10.1007/s00429-017-1445-8

[R10] Engelke DS, Zhang XO, O’Malley JJ, Fernandez-Leon JA, Li S, Kirouac GJ, Beierlein M, and Do-Monte FH (2021). A hypothalamic-thalamostriatal circuit that controls approach-avoidance conflict in rats. Nat. Commun 12, 2517. 33947849 10.1038/s41467-021-22730-y PMC8097010

[R11] Epstein DH, Preston KL, Stewart J, and Shaham Y (2006). Toward a model of drug relapse: An assessment of the validity of the reinstatement procedure. Psychopharmacology (Berl.) 189, 1–16. 17019567 10.1007/s00213-006-0529-6 PMC1618790

[R12] Farrell MR, Schoch H, and Mahler SV (2018). Modeling cocaine relapse in rodents: Behavioral considerations and circuit mechanisms. Prog. Neuropsychopharmacol. Biol. Psychiatry 87 (Pt A), 33–47. 29305936 10.1016/j.pnpbp.2018.01.002 PMC6034989

[R13] Fenno LE, Mattis J, Ramakrishnan C, Hyun M, Lee SY, He M, Tucciarone J, Selimbeyoglu A, Berndt A, Grosenick L, (2014). Targeting cells with single vectors using multiple-feature Boolean logic. Nat. Methods 11, 763–772. 24908100 10.1038/nmeth.2996 PMC4085277

[R14] Fuchs RA, Branham RK, and See RE (2006). Different neural substrates mediate cocaine seeking after abstinence versus extinction training: A critical role for the dorsolateral caudate-putamen. J. Neurosci 26, 3584–3588. 16571766 10.1523/JNEUROSCI.5146-05.2006 PMC1643847

[R15] Giannotti G, Barry SM, Siemsen BM, Peters J, and McGinty JF (2018). Divergent prelimbic cortical pathways interact with BDNF to regulate cocaine-seeking. J. Neurosci 38, 8956–8966. 30185459 10.1523/JNEUROSCI.1332-18.2018 PMC6191522

[R16] Gibson GD, Prasad AA, Jean-Richard-Dit-Bressel P, Yau JOY, Millan EZ, Liu Y, Campbell EJ, Lim J, Marchant NJ, Power JM, (2018). Distinct accumbens shell output pathways promote versus prevent relapse to alcohol seeking. Neuron 98, 512–520.e6. 29656870 10.1016/j.neuron.2018.03.033

[R17] Gong S, Zheng C, Doughty ML, Losos K, Didkovsky N, Schambra UB, Nowak NJ, Joyner A, Leblanc G, Hatten ME, and Heintz N (2003). A gene expression atlas of the central nervous system based on bacterial artificial chromosomes. Nature 425, 917–925. 14586460 10.1038/nature02033

[R18] Hamlin AS, Clemens KJ, Choi EA, and McNally GP (2009). Paraventricular thalamus mediates context-induced reinstatement (renewal) of extinguished reward seeking. Eur. J. Neurosci 29, 802–812. 19200064 10.1111/j.1460-9568.2009.06623.x

[R19] Hearing M, Graziane N, Dong Y, and Thomas MJ (2018). Opioid and psychostimulant plasticity: targeting overlap in nucleus accumbens glutamate signaling. Trends Pharmacol. Sci 39, 276–294. 29338873 10.1016/j.tips.2017.12.004 PMC5818297

[R20] Heinsbroek JA, Neuhofer DN, Griffin WC 3rd, Siegel GS, Bobadilla AC, Kupchik YM, and Kalivas PW (2017). Loss of plasticity in the D2-accumbens pallidal pathway promotes cocaine seeking. J. Neurosci 37, 757–767. 28123013 10.1523/JNEUROSCI.2659-16.2016 PMC5296778

[R21] Hsu DT, Kirouac GJ, Zubieta JK, and Bhatnagar S (2014). Contributions of the paraventricular thalamic nucleus in the regulation of stress, motivation, and mood. Front. Behav. Neurosci 8, 73. 24653686 10.3389/fnbeh.2014.00073 PMC3949320

[R22] Huang AS, Mitchell JA, Haber SN, Alia-Klein N, and Goldstein RZ (2018). The thalamus in drug addiction: From rodents to humans. Philos. Trans. R. Soc. Lond. B Biol. Sci 373, 20170028. 29352027 10.1098/rstb.2017.0028 PMC5790826

[R23] James MH, Charnley JL, Jones E, Levi EM, Yeoh JW, Flynn JR, Smith DW, and Dayas CV (2010). Cocaine- and amphetamine-regulated transcript (CART) signaling within the paraventricular thalamus modulates cocaine-seeking behaviour. PLoS ONE 5, e12980. 20886038 10.1371/journal.pone.0012980 PMC2944892

[R24] Keyes PC, Adams EL, Chen Z, Bi L, Nachtrab G, Wang VJ, Tessier-Lavigne M, Zhu Y, and Chen X (2020). Orchestrating opiate-associated memories in thalamic circuits. Neuron 107, 1113–1123.e4. 32679036 10.1016/j.neuron.2020.06.028 PMC8130576

[R25] Kirouac GJ (2015). Placing the paraventricular nucleus of the thalamus within the brain circuits that control behavior. Neurosci. Biobehav. Rev 56, 315–329. 26255593 10.1016/j.neubiorev.2015.08.005

[R26] Kirouac GJ (2021). The paraventricular nucleus of the thalamus as an integrating and relay node in the brain anxiety network. Front. Behav. Neurosci 15, 627633. 33732118 10.3389/fnbeh.2021.627633 PMC7959748

[R27] Kuhn BN, Klumpner MS, Covelo IR, Campus P, and Flagel SB (2018). Transient inactivation of the paraventricular nucleus of the thalamus enhances cue-induced reinstatement in goal-trackers, but not sign-trackers. Psychopharmacology (Berl.) 235, 999–1014. 29285634 10.1007/s00213-017-4816-1 PMC5871598

[R28] Labouèbe G, Boutrel B, Tarussio D, and Thorens B (2016). Glucose-responsive neurons of the paraventricular thalamus control sucrose-seeking behavior. Nat. Neurosci 19, 999–1002. 27322418 10.1038/nn.4331 PMC4964931

[R29] Li S, and Kirouac GJ (2008). Projections from the paraventricular nucleus of the thalamus to the forebrain, with special emphasis on the extended amygdala. J. Comp. Neurol 506, 263–287. 18022956 10.1002/cne.21502

[R30] Li Y, Wang H, Qi K, Chen X, Li S, Sui N, and Kirouac GJ (2011). Orexins in the midline thalamus are involved in the expression of conditioned place aversion to morphine withdrawal. Physiol. Behav 102, 42–50. 20951152 10.1016/j.physbeh.2010.10.006

[R31] Livneh Y, Ramesh RN, Burgess CR, Levandowski KM, Madara JC, Fenselau H, Goldey GJ, Diaz VE, Jikomes N, Resch JM, (2017). Homeostatic circuits selectively gate food cue responses in insular cortex. Nature 546, 611–616. 28614299 10.1038/nature22375 PMC5577930

[R32] Lobo MK, and Nestler EJ (2011). The striatal balancing act in drug addiction: Distinct roles of direct and indirect pathway medium spiny neurons. Front. Neuroanat 5, 41. 21811439 10.3389/fnana.2011.00041 PMC3140647

[R33] Lopes G, Bonacchi N, Frazão J, Neto JP, Atallah BV, Soares S, Moreira L, Matias S, Itskov PM, Correia PA, (2015). Bonsai: An event-based framework for processing and controlling data streams. Front. Neuroinform 9, 7. 25904861 10.3389/fninf.2015.00007 PMC4389726

[R34] Matzeu A, and Martin-Fardon R (2018). Drug seeking and relapse: New evidence of a role for orexin and dynorphin co-transmission in the paraventricular nucleus of the thalamus. Front. Neurol 9, 720. 30210441 10.3389/fneur.2018.00720 PMC6121102

[R35] McDevitt DS, and Graziane NM (2019). Timing of morphine administration differentially alters paraventricular thalamic neuron activity. eNeuro 6, ENEURO.0377–19.2019. 10.1523/ENEURO.0377-19.2019 PMC6920517 31801741

[R36] McGinty JF, and Otis JM (2020). Heterogeneity in the paraventricular thalamus: The traffic light of motivated behaviors. Front. Behav. Neurosci 14, 590528. 33177999 10.3389/fnbeh.2020.590528 PMC7596164

[R37] McNally GP (2021). Motivational competition and the paraventricular thalamus. Neurosci. Biobehav. Rev 125, 193–207. 33609570 10.1016/j.neubiorev.2021.02.021

[R38] Millan EZ, Ong Z, and McNally GP (2017). Paraventricular thalamus: Gateway to feeding, appetitive motivation, and drug addiction. Prog. Brain Res 235, 113–137. 29054285 10.1016/bs.pbr.2017.07.006

[R39] National Research Council (US) Committee for the Update of the Guide for the Care and Use of Laboratory Animals (2011). Guide for the Care and Use of Laboratory Animals, Eighth Edition (National Academies Press). 21595115

[R40] Otis JM, Namboodiri VM, Matan AM, Voets ES, Mohorn EP, Kosyk O, McHenry JA, Robinson JE, Resendez SL, Rossi MA, and Stuber GD (2017). Prefrontal cortex output circuits guide reward seeking through divergent cue encoding. Nature 543, 103–107. 28225752 10.1038/nature21376 PMC5772935

[R41] Pascoli V, Terrier J, Espallergues J, Valjent E, O’Connor EC, and Lüscher C (2014). Contrasting forms of cocaine-evoked plasticity control components of relapse. Nature 509, 459–464. 24848058 10.1038/nature13257

[R42] Paxinos G, and Watson C (2007). The Rat Brain in Stereotaxic Coordinates (Academic Press). 10.1016/0165-0270(80)90021-7 6110810

[R43] Penzo MA, and Gao C (2021). The paraventricular nucleus of the thalamus: An integrative node underlying homeostatic behavior. Trends Neurosci. 44, 538–549. 33775435 10.1016/j.tins.2021.03.001 PMC8222078

[R44] Penzo MA, Robert V, Tucciarone J, De Bundel D, Wang M, Van Aelst L, Darvas M, Parada LF, Palmiter RD, He M, (2015). The paraventricular thalamus controls a central amygdala fear circuit. Nature 519, 455–459. 25600269 10.1038/nature13978 PMC4376633

[R45] Peters J, Kalivas PW, and Quirk GJ (2009). Extinction circuits for fear and addiction overlap in prefrontal cortex. Learn. Mem 16, 279–288. 19380710 10.1101/lm.1041309 PMC4527308

[R46] Pickens CL, Airavaara M, Theberge F, Fanous S, Hope BT, and Shaham Y (2011). Neurobiology of the incubation of drug craving. Trends Neurosci. 34, 411–420. 21764143 10.1016/j.tins.2011.06.001 PMC3152666

[R47] Reiner DJ, Fredriksson I, Lofaro OM, Bossert JM, and Shaham Y (2019). Relapse to opioid seeking in rat models: Behavior, pharmacology and circuits. Neuropsychopharmacology 44, 465–477. 30293087 10.1038/s41386-018-0234-2 PMC6333846

[R48] Roura-Martínez D, Ucha M, Orihuel J, Ballesteros-Yáñez I, Castillo CA, Marcos A, Ambrosio E, and Higuera-Matas A (2020). Central nucleus of the amygdala as a common substrate of the incubation of drug and natural reinforcer seeking. Addict. Biol 25, e12706. 30623520 10.1111/adb.12706

[R49] Shin G, Gomez AM, Al-Hasani R, Jeong YR, Kim J, Xie Z, Banks A, Lee SM, Han SY, Yoo CJ, (2017). Flexible near-field wireless opto-electronics as subdermal implants for broad applications in optogenetics. Neuron 93, 509–521.e3. 28132830 10.1016/j.neuron.2016.12.031 PMC5377903

[R50] Spanagel R (2017). Animal models of addiction. Dialogues Clin. Neurosci 19, 247–258. 29302222 10.31887/DCNS.2017.19.3/rspanagel PMC5741108

[R51] Steketee JD, and Kalivas PW (2011). Drug wanting: Behavioral sensitization and relapse to drug-seeking behavior. Pharmacol. Rev 63, 348–365. 21490129 10.1124/pr.109.001933 PMC3082449

[R52] Tye KM, and Deisseroth K (2012). Optogenetic investigation of neural circuits underlying brain disease in animal models. Nat. Rev. Neurosci 13, 251–266. 22430017 10.1038/nrn3171 PMC6682316

[R53] Tzschentke TM (2007). Measuring reward with the conditioned place preference (CPP) paradigm: Update of the last decade. Addict. Biol 12, 227–462. 17678505 10.1111/j.1369-1600.2007.00070.x

[R54] Venniro M, Caprioli D, and Shaham Y (2016). Animal models of drug relapse and craving: From drug priming-induced reinstatement to incubation of craving after voluntary abstinence. Prog. Brain Res 224, 25–52. 26822352 10.1016/bs.pbr.2015.08.004

[R55] Venniro M, Caprioli D, Zhang M, Whitaker LR, Zhang S, Warren BL, Cifani C, Marchant NJ, Yizhar O, Bossert JM, (2017). The anterior insular cortex/central amygdala glutamatergic pathway is critical to relapse after contingency management. Neuron 96, 414–427.e8. 29024664 10.1016/j.neuron.2017.09.024 PMC5687288

[R56] Venniro M, Russell TI, Ramsey LA, Richie CT, Lesscher HMB, Giovanetti SM, Messing RO, and Shaham Y (2020). Abstinence-dependent dissociable central amygdala microcircuits control drug craving. Proc. Natl. Acad. Sci. USA 117, 8126–8134. 32205443 10.1073/pnas.2001615117 PMC7148559

[R57] Yasoshima Y, Scott TR, and Yamamoto T (2007). Differential activation of anterior and midline thalamic nuclei following retrieval of aversively motivated learning tasks. Neuroscience 146, 922–930. 17412515 10.1016/j.neuroscience.2007.02.044

[R58] Zhou K, and Zhu Y (2019). The paraventricular thalamic nucleus: A key hub of neural circuits underlying drug addiction. Pharmacol. Res 142, 70–76. 30772461 10.1016/j.phrs.2019.02.014

[R59] Zhou K, Zhu L, Hou G, Chen X, Chen B, Yang C, and Zhu Y (2021). The contribution of thalamic nuclei in salience processing. Front. Behav. Neurosci 15, 634618. 33664657 10.3389/fnbeh.2021.634618 PMC7920982

[R60] Zhu Y, Wienecke CF, Nachtrab G, and Chen X (2016). A thalamic input to the nucleus accumbens mediates opiate dependence. Nature 530, 219–222. 26840481 10.1038/nature16954 PMC4814115

[R61] Zhu Y, Nachtrab G, Keyes PC, Allen WE, Luo L, and Chen X (2018). Dynamic salience processing in paraventricular thalamus gates associative learning. Science 362, 423–429. 30361366 10.1126/science.aat0481 PMC6521722

