# Supplementary material for: Extinction blunts paraventricular thalamic contributions to heroin relapse
Source: Cell Rep. Author manuscript; Available in PMC 2021 Sep 5. (PMC8418780; doi:10.1016/j.celrep.2021.109605)
Supplement: 1 [file NIHMS1735790-supplement-1.pdf]

Cell Reports, Volume *36*

Supplemental information

Extinction blunts paraventricular thalamic contributions to heroin relapse

Giuseppe Giannotti, Sheng Gong, Nicholas Fayette, Jasper A. Heinsbroek, James E. Orfila, Paco S. Herson, Christopher P. Ford, and Jamie Peters

Supplemental information

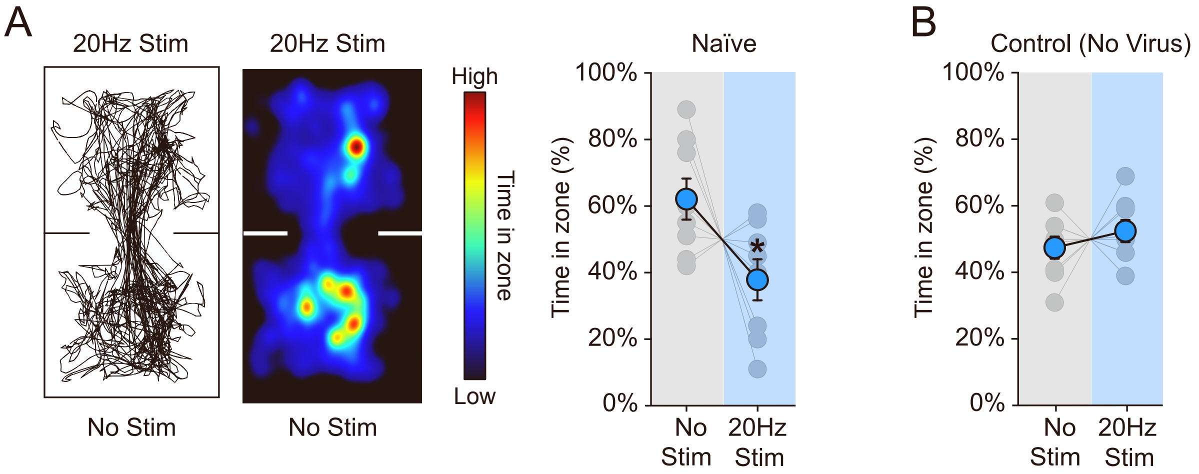

Figure S1: Optogenetic activation of PVT→NAc pathway induces aversion in naïve animals (related to Figure 2 and Figure 3). A) Example trace (left) and heatmap (middle) of real-time CPA test. Optogenetic activation (20Hz, 20ms) of the PTV→NAc pathway induced real-time CPA in naïve animals. **B)** Stimulation of PVT (20Hz, 20ms) in the absence of ChR2 has no effect in a real-time CPA test. * $p<0.05$ vs. no stim condition. Data are shown as mean ± SEM.

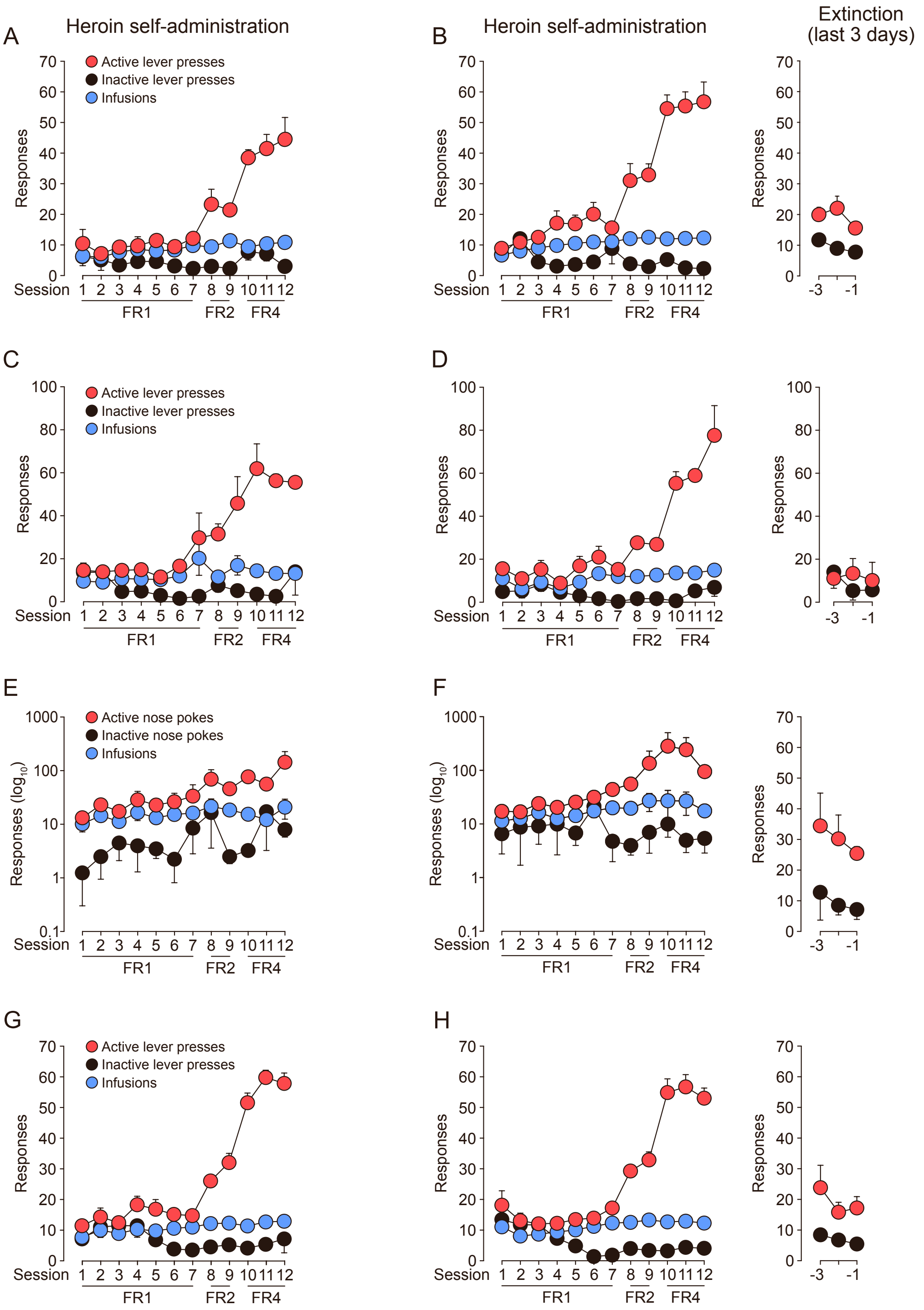

Figure S2. Heroin self-administration and extinction (related to Figure 2, Figure 3, Figure 4, Figure 5, and Figure S4). Heroin self-administration of the abstinence **(A)** and the extinction group **(B, left)** for the optogenetic experiments. Panel **B (right)** shows last 3 days of extinction training. Heroin self-administration of the abstinence **(C)** and the extinction group **(D, left)** for the field post-synaptic potential experiments. Panel **D (right)** shows last 3 days of extinction training. Heroin self-administration of the abstinence **(E)** and the extinction group **(F, left)** for the LTD experiments in the D2-eGFP mice. Panel **F (right)** shows last 3 days of extinction. Heroin self-administration of the abstinence **(G)** and the extinction group **(H, left)** for the chemogenetic experiments. Panel **H (right)** shows last 3 days of extinction training. Data are shown as mean ± SEM.

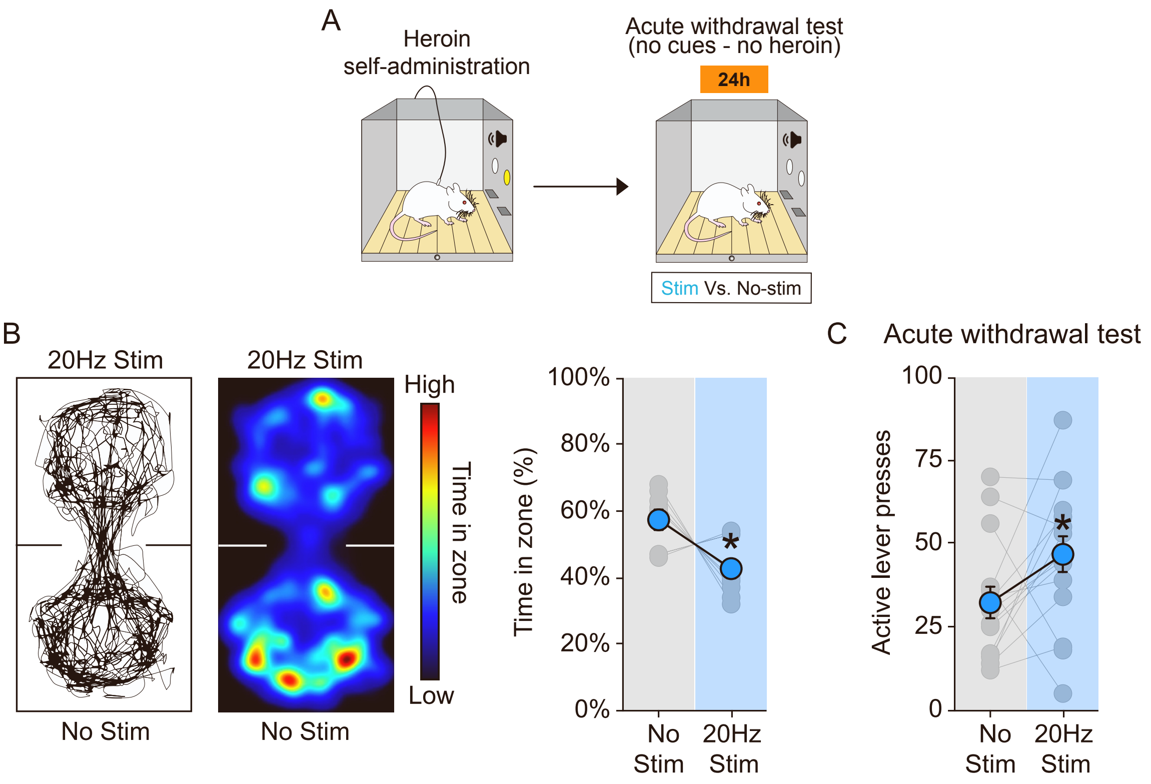

Figure S3: Optogenetic activation of the PVT→NAc pathway induces aversion and drives heroin seeking during early withdrawal from heroin self-administration (related to Figure 3). A) Timeline employed for the behavioral experiment. Optogenetic activation (20Hz, 20ms) of the PVT→NAc pathway induces aversion **(B)** and drives heroin seeking **(C)** during early (24 h) withdrawal from heroin self-administration. * $p<0.05$ vs. no stim condition. Data are shown as mean ± SEM.

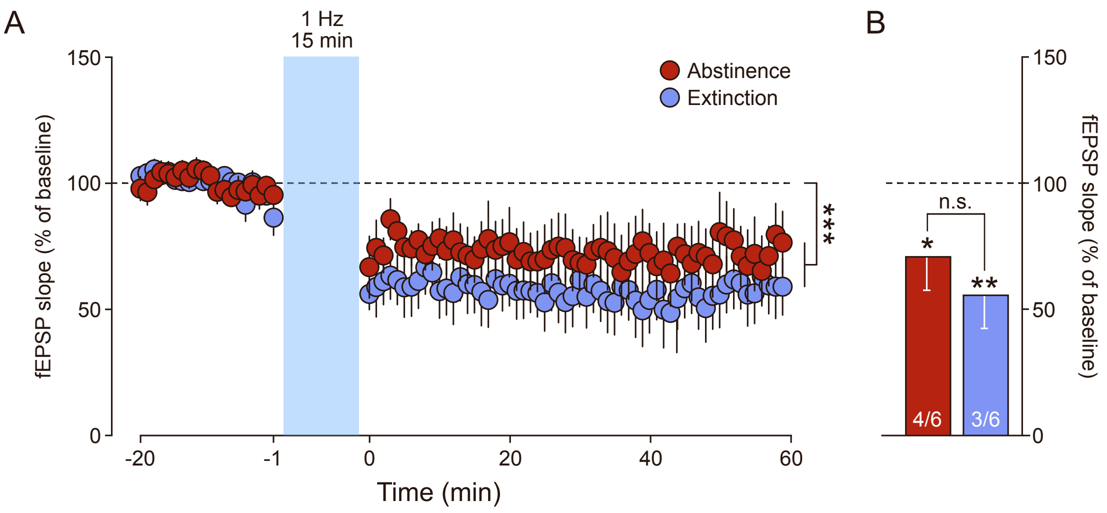

Figure S4: LTD stimulation protocol of ChR2-expressing PVT terminals in the NAc drives synaptic plasticity after extinction and abstinence from heroin self-administration (related to figure 4). A) Time course of optogenetic evoked field excitatory post-synaptic potential (fEPSP) slopes in NAc slices from abstinence and extinction animals (*** $p<0.001$, main effect of the time). **B)** Optogenetic stimulation (1 Hz - 900 pulses) of ChR2-expressing PVT terminals in the NAc induces LTD after abstinence and extinction from heroin self-administration (average of the last 20 min post-LTD; * $p<0.05$, ** $p<0.01$ vs baseline). Data are shown as mean ± SEM.
